# Detection of Magnetic Field Intensity Gradient by Homing Pigeons (*Columba livia*) in a Novel “Virtual Magnetic Map” Conditioning Paradigm

**DOI:** 10.1371/journal.pone.0072869

**Published:** 2013-09-09

**Authors:** Cordula V. Mora, Verner P. Bingman

**Affiliations:** Department of Psychology and J.P. Scott Center for Neuroscience, Mind and Behavior, Bowling Green State University, Bowling Green, Ohio, United States of America; The Australian National University, Australia

## Abstract

It has long been thought that birds may use the Earth's magnetic field not only as a compass for direction finding, but that it could also provide spatial information for position determination analogous to a map during navigation. Since magnetic field intensity varies systematically with latitude and theoretically could also provide longitudinal information during position determination, birds using a magnetic map should be able to discriminate magnetic field intensity cues in the laboratory. Here we demonstrate a novel behavioural paradigm requiring homing pigeons to identify the direction of a magnetic field intensity gradient in a “virtual magnetic map” during a spatial conditioning task. Not only were the pigeons able to detect the direction of the intensity gradient, but they were even able to discriminate upward versus downward movement on the gradient by differentiating between increasing and decreasing intensity values. Furthermore, the pigeons typically spent more than half of the 15 second sampling period in front of the feeder associated with the rewarded gradient direction indicating that they required only several seconds to make the correct choice. Our results therefore demonstrate for the first time that pigeons not only can detect the presence and absence of magnetic anomalies, as previous studies had shown, but are even able to detect and respond to changes in magnetic field intensity alone, including the directionality of such changes, in the context of spatial orientation within an experimental arena. This opens up the possibility for systematic and detailed studies of how pigeons could use magnetic intensity cues during position determination as well as how intensity is perceived and where it is processed in the brain.

## Introduction

Since their domestication several thousand years ago, homing pigeons (*Columba livia f. domestica)* have demonstrated countless times their impressive ability to home to their loft from distant and unfamiliar places. Thus, pigeons have become one of the main model species for studying avian navigation in general and the use of the Earth's magnetic field during homing in particular.

It has been well established that pigeons possess an innate magnetic compass for direction finding (“compass”-step) [1] and some progress has been made in recent years in relation to identifying a putative receptor system for magnetic compass perception [2]. More controversial has been the question as to whether pigeons, and birds in general, use magnetic field intensity for position determination (“map”-step) during navigation, especially given that the discovery of an avian magnetoreceptor for a magnetic intensity seems as elusive as ever [3]. The likely existence of a magnetic map has been, however, demonstrated in the last decade in other animal groups with evidence for magnetic positioning-fixing having been accumulated for such a diverse array of species as lobsters [4], newts [5]–[6], marine turtles [7]–[8], and migratory birds [9]–[10].

Early evidence for the potential existence of a “magnetic map” in homing pigeons comes from the observation that spatial (anomalies) and temporal (solar flares) disturbances to the Earth's magnetic field can lead to temporary disorientation of pigeons [11]–[12] even under sunny conditions, when the sun compass is available for direction finding. Magnetic field intensity could theoretically provide both latitudinal and longitudinal positional information [13]–[14] and recent studies have demonstrated indirectly associations of vanishing bearings and GPS-tracks of pigeons with the magnetic intensity contour lines at the release site at least for sites located in Germany and New Zealand [15]–[16]. This is indirectly supported by additional evidence for birds and other animals sensing magnetic intensity (e.g., [17]–[19]).

Any progress in unraveling a possible magnetic map in homing pigeons has, however, been hampered by two factors. Firstly, despite the development of GPS data-loggers small enough to be carried by homing pigeons, the difficulty with field studies remains that it has not yet been possible to achieve a direct connection between an experimental treatment aimed at disrupting a potential magnetic map mechanism and a behavioural effect being observed. This is because other sensory cues may be available to the pigeon for position determination during homing [20]. Consequently, to test experimentally the existence of a magnetic map, all other sensory cues but magnetic ones should be excluded. Yet it is highly unlikely that a pigeon that has been made anosmic (olfactory nerve sectioning), deaf to infrasound (ear plugs) and blind to landscape features (frosted lenses) would still be willing to attempt to home. The situation is further complicated by the fact that what sensory cue(s) is or are used for position determination may depend on the location the pigeon is raised in during a navigationally formative period. That is, even though a magnetic map may be theoretically globally sufficient for position determination, in areas with other salient cues, such as strong odour gradients related to geography (e.g., [21]–[22]), pigeons may favour alternative cues during homing when available and when the pigeons were previously exposed to them.

Secondly, in contrast to recent discoveries related to avian magnetic compass perception, no real progress has been made in advancing physiological evidence for a candidate magnetic intensity receptor in birds that has been directly linked to a behavioural output since early works with a migratory bird, the bobolink *Dolichonyx oryzivorus* (e.g., [17] & [23]). Even the to-date most advanced model of a candidate magnetic intensity receptor system (e.g., [24]), which proposed iron-based structures located at three bilateral locations in the upper beak of homing pigeons, was solely based on anatomical studies and its feasibility has very recently been seriously called into question [25]. The only study of a candidate magnetic intensity receptor, which linked behaviour, structure and function, was completed in rainbow trout (a migratory salmon species) more than a decade ago [26].

Our goal was to test for the first time whether homing pigeons are able to detect and respond to magnetic intensity changes in the context of spatial orientation within an experimental arena (with close to constant magnetic inclination). We decided to use a conditioning approach as conditioning studies in the laboratory permit control of all sensory cues experienced by the animal, while at the same time ensuring the animal's motivation to perform the behavioural task is maintained. Using conditioning techniques, it has been possible to demonstrate general magnetic sensitivity, that is responses to changes in both magnetic intensity and inclination, in several species including homing pigeons (honey bees *Apils mellifera* [27]; yellow-fin tuna *Thunnus albacares* [28]; rainbow trout *Oncorhynchus mykiss* [26]; homing pigeons *Columba livia* [29] & [30]; short-tailed stingray *Dasyatis brevicaudata* [31]; chickens *Gallus gallus domesticus* [32]; zebrafish *Danio rerio* [33]; zebra finches *Taeniopygia guttata* [34]).

What traditional conditioning approaches are not able to provide, however, is an understanding of whether and, if so, how magnetic cues are used during spatial orientation. Whilst the experimental approach presented here still does not yet allow us to answer the question as to exactly how pigeons use magnetic cues for position determination, it for the first time allowed us to ask what types of magnetic intensity information pigeons are able to perceive, which would provide some insight into what magnetic cues are available to a pigeon at the sensory level for a proposed magnetic map. For this purpose we combined conditioning procedures with the traditional orientation arena approach previously used for measuring spontaneous orientation responses in sea turtles in the presence of specific magnetic field values (e.g., [35]). Thus, this approach was intended to go beyond the traditional discrimination of the presence versus absence of a magnetic field anomaly varying both in magnetic field intensity and inclination that had been previously demonstrated for homing pigeons [29].

In this study, we therefore varied magnetic field intensity in a novel “virtual magnetic intensity map”, which consisted of a simple magnetic field intensity gradient. The pigeons were required to use the magnetic intensity cues to solve a spatial orientation task in the absence of all other sensory cues. Specifically they were required to identify the spatial orientation of the gradient in relation to four available feeders with the directionality at each feeder either being associated with rapidly increasing, rapidly decreasing, or unchanging magnetic field intensity. The implications of our findings as related to magnetic field intensity perception in homing pigeons as well as the future potential for this novel approach for studying the use of magnetic intensity cues during position determination are discussed.

## Materials and Methods

### Experimental Subjects

Six adult homing pigeons (*Columba livia livia f. domestica*), three males and three females, all more than one year old and with previous homing experience, were housed individually at Bowling Green State University in Bowling Green, Ohio, United States. They were provided with water *ad libitum* and maintained between 85 and 87% free-feeding body weight to ensure motivation during the conditioning task.

### Experimental Setup

**Figure 1:**
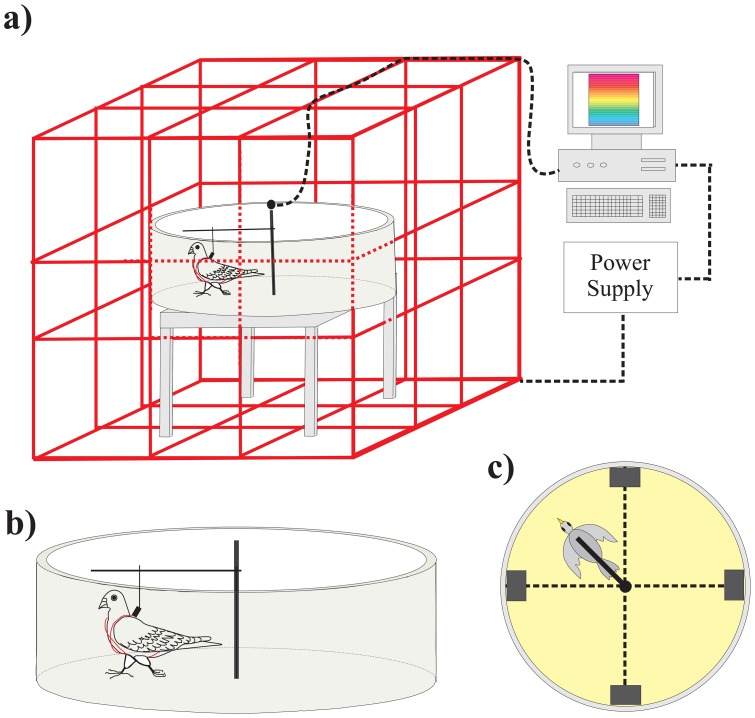
Experimental setup for virtual magnetic map conditioning paradigm (not drawn to scale). a) Circular orientation arena (diameter 110 cm) surrounded by three-axis coils system (red lines; adapted from [24]), which generated a spatially uniform magnetic field intensity cue throughout the entire arena. This type of magnetic cue is in contrast to spatially variable magnetic anomalies used by past conditioning studies (e.g., [17]). Magnetic field intensity in the arena was controlled in real time via customized MVR (Magnetic Virtual Reality) software based on the position of the pigeon over time within a virtual magnetic intensity map. Note that the arena's four feeders-response units are not shown for clarity. b) Pigeon walking in arena whilst attached via harness (red) to horizontal tracker arm (adapted from previous sea turtle studies [23]), with tracker arm orientation in the arena detected by angular decoder every 200 ms. Note that the arena's four feeders-response units are not shown for clarity. c) Top view of arena showing pigeon attached to horizontal tracker arm as well as position of four feeder-response units (grey rectangles), each with a pecking key above an automated food reservoir, located around the periphery of the circular arena in the four cardinal directions (geographic North, South, East and West; dashed lines).

All experiments were conducted in a circular arena (diameter 110 cm; wall height 38 cm) situated centrally atop cinder blocks inside a 3-axis magnetic coil system (Figure 1a; see text below). Pigeons were individually harnessed to a horizontal tracker arm (Figure 1b). The harness consisted of two 1.5 cm wide strips of fabric cat collars sewn together in the shape of an “X” with a clip attached at the joint and resting between the wings on the pigeon's back for attachment to the tracker arm.

The horizontal tracker arm was attached to a central, vertical shaft in the arena (Figure 1b). The pigeon was able to walk freely around the periphery of the arena in either direction at a distance of 35 cm (point of attachment of harness on pigeon's back to tracker arm) from the center of the arena. An angular decoder located at the base of the shaft recorded the pigeon's position to the nearest degree once every 200 millisecond. Four automated feeder-response units were situated against the wall of the circular arena aligned with the four cardinal directions in the test room (geographic North (N), South (S), East (E), and West (W); Figure 1c). Each feeder-response unit contained an illuminated food magazine with food pellets (Purina® Check Pigeon Chow pellets) that could be made accessible to the pigeon and a response pecking key above the food magazine that could be illuminated. Each feeder's food magazine was raised and lowered by compressed air to avoid any localized distortions to the magnetic field typically associated with motor-driven feeders. A pigeon was easily able to reach each of the feeder-response units with its beak as the units protruded into the arena by 7 cm. Furthermore, the harness clip attaching it to the tracker arm was able to rotate and slightly move, and the pigeon additionally was able to extend its neck from its harnessed position toward the feeder. An incandescent white light was mounted centrally above the circular arena as the trial light. The behaviour of the pigeon in the arena was monitored via a centrally-mounted close-circuit video camera viewing the arena from above.

The 3-axis magnetic coil system (four 240×240 cm square coils per axis with a coil spacing of 89/62/89 cm; coil winding ratio of 26:11:11:26; 14 AWG, PVC-insulated copper coil wire, aluminium frame, adapted from [36]; Figure 1a) was powered by three power supplies (BK Precision, Model 9123A, 0–30V/0–5A Single Output Programmable DC power supply with constant current output), one assigned to each axis (x, y, and z) of the coil system. This coil system was able to generate a sphere-shaped area in the center of the coils, approximately the size of the diameter of the experimental arena, within which the generated magnetic field was very uniform, albeit not perfectly uniform as is typically the case with this type of coil system. That is, the magnetic field vector was the very similar in terms of length (intensity) and spatial orientation (inclination and declination) for all spatial points inside this “bubble”. By changing independently the current output to each of the three coil axes, we were able to either increase or decrease the magnetic field intensity *in real time* and relatively *uniformly* throughout the entire experimental arena (note that this is a different type of magnetic field manipulation from generating a spatial gradient from one side of the arena to the other or creating a localized magnetic anomaly as in [29]). A white noise generator positioned next to the coil system masked any potential humming noise emanating from the coil system. The power supplies and associated relays were located in a control room adjacent to the room containing the coil system. The coil wiring remained cool to the touch throughout the conditioning sessions.

**Figure 2:**
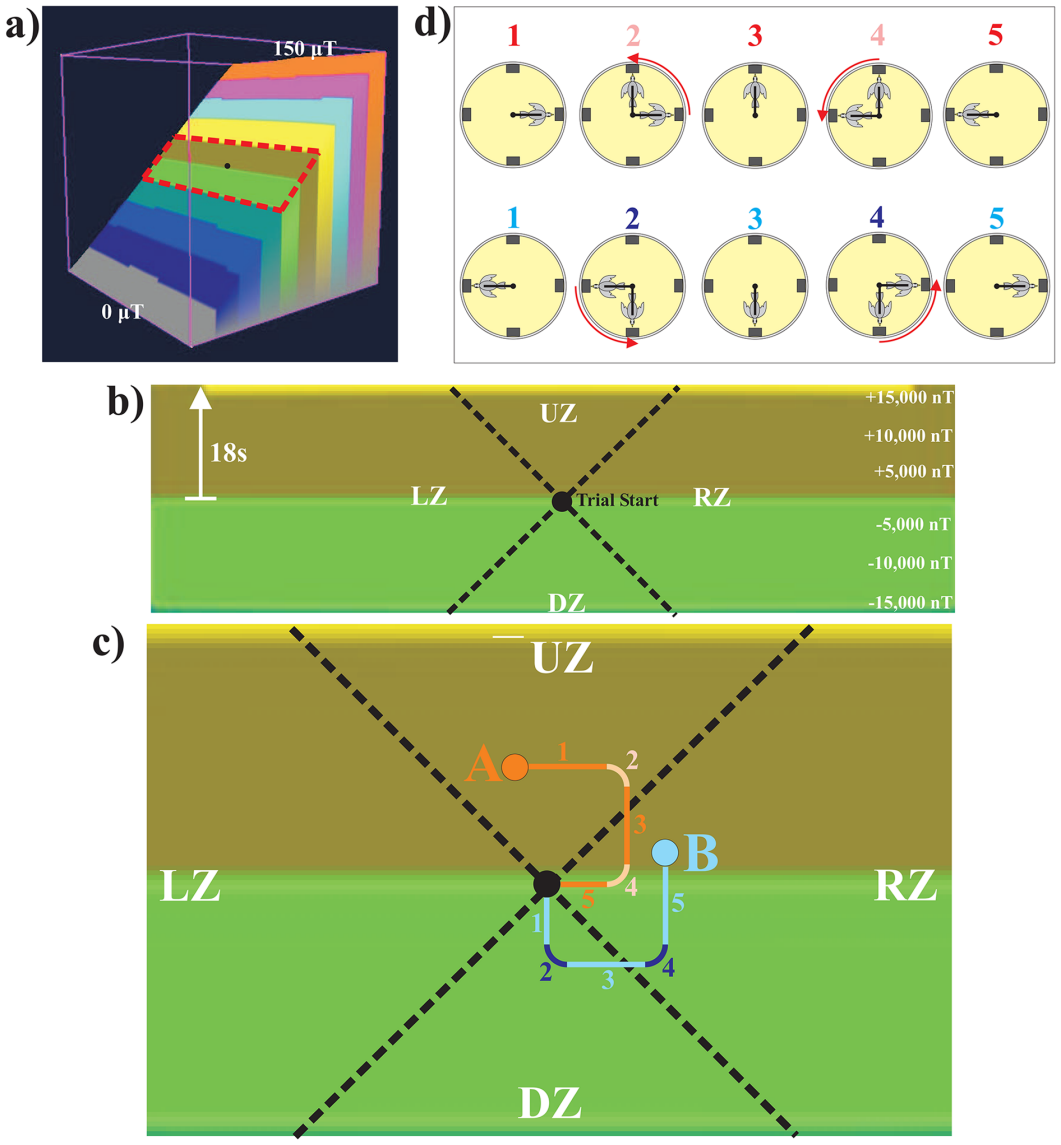
Virtual magnetic intensity map (VMI-map) and generation of individual tracks by pigeons inside this map. a) Angled side view of VMI-map consisting of simple slope of steadily increasing magnetic intensity with inclination and declination held constant at background level. This map does not represent a spatial gradient within the arena from one side to the other, but rather a dynamic virtual environment comparable to that presented visually to players of first-person-perspective computer games whilst moving through virtual environments (see main text for more details). Note that coloured bands on the map do not represent a step-wise increase in magnetic intensity, but were only used to aid visual comparison of a pigeon's tracks during a session. Orientation of gradient direction within the experimental room (i.e., which one of the four feeders was associated with upward-gradient movement) was based on a pseudo-random schedule for each trial. Pigeon was “released” at the start of the trial in the center of the VMI-map (black dot) and magnetic field intensity changes experienced within a 15-s trial were limited in this study to an area of less than ±15,000 nT (red dashed line; enlarged in Figure 2b). b) Enlarged section of VMI-map showing operational range for this study. When the pigeon faced the feeder associated with either the up- or down-gradient movement in map, magnetic intensity change occurred at a maximum speed of 833 nT per second with one coloured band being crossed in 18 seconds. Thus, the maximum increase or decrease in intensity experienced for a 15-s trial, achieved by the pigeon sitting in front of either of the feeders associated with magnetic intensity increases or decreases respectively, was ±12,500 nT. Point of the pigeon's “release” at the start of the 15-s trial (small black circle) and subdivision (black dashed lines) of map into four zones (up-gradient zone (UZ), down-gradient zone (DZ), left-side zone (LZ), and right-side zone (RZ)) are shown. c) Frontal view of VMI-map with two simulated tracks (red and blue lines). During a trial, the pigeon was “released” in the center of the map (small black circle) and its path in the VMI-map plotted in real time as a track. Note that magnetic field intensity was adjusted in real time uniformly throughout the entire arena based on the pigeon's current track position within the virtual map. That is, as the track was plotted in the map based on which direction in the arena the pigeon's tracker arm faced over time, the pigeon was exposed to the magnetic field intensity in accordance with its current position in the VMI-map. Sitting still in front of a feeder resulted in straight track lines, while movement between feeders resulted in angular or curved track lines depending on the speed of rotation by the pigeon. At the end of a 15 s sampling period the pigeon was allowed to choose one feeder by pecking that feeder's illuminated pecking key with only responses at the feeder associated with either up-gradient movement (up-gradient group) or down-slope movement (down-gradient group) as indicated by increasing or decreasing intensity, respectively, being rewarded with food. Each tracks terminal point at the end of the 15-s sampling period was scored to fall into one of four zones (see above). For the red track the up-gradient orientation was associated with the North feeder and for the blue track with the East feeder. Numbers next to each track identify the differently coloured track segments linked with the pigeon's orientation in the arena in Figure 2d. Note that while the red track terminated at the end of the 15-s trial at “A” in the up-gradient zone, the pigeon had switched for its final response to a feeder associated with sideways movement in the map, and thus its choice was unrewarded. The blue track terminated at “B” in the right-side zone, but the pigeon had switched for its final choice to the feeder associated with up-gradient movement (increasing intensity) in the map. Such a choice would have been rewarded for a bird in the up-gradient group, but not the down-gradient group. d) Diagrams illustrating the pigeon's position or movement between positions (red arrow) in the arena for the time of the red (top row) and blue (bottom row) track's individual segments seen in Figure 2c. The four feeder-response units are shown as grey rectangles.

The amount of current supplied to each coil axis was fully automated via custom-written “Magnetic Virtual Reality” (MVR) software. The MVR software generated in the computer a “virtual magnetic intensity map” (VMI-map) consisting of a simple magnetic intensity gradient ranging from 0 μTesla (μT) to 150 μT (approximately 3 times the local magnetic field intensity of 47,300 μT (see below); Figure 2a), while at the same time holding magnetic inclination (within ±2°) and declination (within ±8°) almost constant. Even though the rate of increase in magnetic intensity was the same from the bottom to the top of the VMI-map, on the computer screen and thus in our figures the map was visually divided into ten coloured bands of equal width to facilitate visualization and comparison of the pigeons' tracks in the map. The speed at which the pigeon “moved through” the map, in real time, during a trial (see text below for more detail) was set for this study such that it would take a given trial's track 18 seconds to transit through one of the coloured bands when moving straight up or down the intensity gradient within the computer-based map (equivalent to a ±15,000 nT intensity change). Consequently, the pigeon experienced a maximum magnetic intensity change of 12,500 nT during the 15 s sampling period (at a maximum speed of 833 nT/s; Figure 2b), which is approximately 1/4 of the local magnetic background intensity. Thus, the operational range (red dashed line in Figure 2a) of the VMI-map for tracks generated during the 15 s sampling period was considerably smaller than the full range of the map available for future studies.

The background field and the magnetic field parameters generated by the coil system were characterized with a FVM handheld 3-axis vector fluxgate magnetometer (Meda Inc.) at the head height of a walking pigeon and at a distance of 30 cm from the center of the arena. Due to structural steel and electrical circuits in the walls of the experimental room, the background magnetic intensity varied around the periphery of the arena along a SW to NE gradient (mean 47,300 nT ±330 SE with values ranging from 45,350 to 49,100 nT). Background magnetic inclination and declination varied between +61.9° and +68.2° (Mean +65.0°±0.4 SE) and +4.8° and +24.5° (Mean +17.6°±1.2 SE), respectively. In contrast to the variations in the background field, the coil system itself generated a magnetic field vector whose intensity varied, as expected, strongly during the trial when the birds moved up or down the gradient, but which had little spatial variation throughout the arena (mean values throughout the arena for settings along the magnetic gradient typically were associated with a standard error value of around 50 nT). At the same time there were only minimal temporal and spatial inclination and declination changes when background variations were subtracted. Changes in inclination and declination at each feeder location throughout the 15 s trial during sessions with parallel coil settings (see below) ranged only between 0.00° to 0.34° and 0.07° to 1.13° respectively (Figures S1 & S3). The differences between the maximum and minimum magnetic inclination and declination values for 25 locations where measurements were taken throughout the arena (see also figure caption of Figures S5 & S6) averaged 1.43° (±0.21° SE) and 6.00° (±0.99° SE) for inclination and declination, respectively.

### Pre-Training Procedure

The pigeons were familiarized with the harness initially by being fed in their home cage whilst wearing the harness. During pre-training sessions, they were next attached via the harness to the tracker arm in the experimental arena with food placed on the floor in the locations where the four feeders would be later situated. Once the pigeons had acclimatized to being attached to the tracker arm and ate freely in the arena, the four feeders were added to the arena as described above. Pigeons were then familiarized with the food magazine being raised and lowered in a pseudo-random order on each of the four feeders to allow food access for 10 s. Finally, the pigeons were required to peck a feeder's illuminated pecking key before the feeder's food magazine was raised, with pecks being detected by a micro-switch situated behind the key and registered by the MVR software. Pecking keys were made available in a pseudorandom order during each pre-training session's 16 trials to avoid any response biases.

Due to the complexity of the conditioning task to be learned, with four feeders being distributed around the periphery of the arena and a dynamically changing magnetic stimulus, the pigeons were next exposed for five sessions (16 trials per session) to the reinforcement contingencies associated with the VMI-map (Figure 2). That is, the pigeons received pre-training for the exact spatial orientation task they were later required to perform for data collection. This was achieved by manually aligning the gradient direction of either increasing or decreasing magnetic intensity (depending on whether a bird belonged to the group rewarded for choosing the feeder associated with increasing or decreasing intensity respectively) with the nearest feeder to the pigeon. The pigeon was allowed to experience the increasing or decreasing field intensity for 15 s before the correct feeder's peck light was switched on. Pecking this key resulted in a food reward. For a certain number of trials in each of these sessions, it was ensured that the pigeon experienced the stimulus that was the correct one for the feeder the pigeon was positioned nearest to during the trial to form an association between stimulus and correct feeder choice. Over the course of the five sessions, the number of trials during which assistance was given to the bird as to which feeder was the correct one for a given stimulus presentation was gradually reduced. At the same time, the number of trials during which the pigeon was allowed to make its own choices was increased. Therefore, by the end of pre-training the pigeons were already very familiar with the basic discrimination task, which is why no learning curve was observed during the first experimental conditioning series.

### Magnetic Conditioning Procedure

Magnetic conditioning sessions were conducted with individual pigeons. Each session consisted of 32 discrete trials. At the start of a session, the pigeon was harnessed to the tracker arm in the darkened arena with its placement at the periphery of the arena determined by a pseudorandom schedule (see below).

At the start of each trial, as indicated by the trial light being switched on, the pigeon was “released” in the center of the VMI-map (small black circle; Figure 2a&b), in other words, at an intensity value of between 82,300 nT (at South feeder) and 84,750 nT (at North feeder). During a sampling period (15 s), measured with a stop watch, the pigeon was able to move freely around the periphery of the arena. During this time, the MVR software plotted the position of the pigeon in real time within the VMI-map as a track depending on the pigeon's position around the periphery of the arena over time. The current output to the three coil axes was simultaneously and continually adjusted to generate uniformly throughout the entire arena the magnetic intensity value of the pigeon's current position in the map. The aim was to simulate to the pigeon movement within the map over time, *the important point being that intensity change was determined by a pigeon's moment to moment position in the arena (as indicated by the tracker arm position) and not directly related to movement by the pigeon or the pigeon's orientation*. Thus, this approach is analogous to players of some popular computer games visually moving in first-person-perspective through virtual environments. In such gaming systems, a computer keeps track of the player's spatial orientation over time and simulates visually in real time movement through the virtual environment to the player based on the player's current position and orientation in the environment.

As such, the pigeon was experiencing a dynamic virtual magnetic intensity environment with the intensity presented to the pigeon changing over time based on the position of the pigeon's tracker arm, and thus, where in the map the pigeon currently was located (a slight spatial component was added due to the small intensity gradient in the background field across the experimental arena). The orientation of the map's intensity slope in relation to the four feeders, i.e., which feeder was associated with up-gradient, down-gradient and left/right sideway movements in the map, was changed for each trial based on a pseudorandom schedule to avoid the pigeons using any visual cues to solve the spatial conditioning task. Each session consisted of 32 trials divided into four 8-trial blocks. Thus, to avoid any visual cues being used to identify the correct feeder choice, for each 8-trial block, the feeder for the up-gradient direction was randomly selected (without replacement) from an array of directions that consisted of two of each of the four possible cardinal directions (North, East, South, and West). Thus, the order of stimulus presentation for an 8-trial block drawn from this array could, for example, look like “E, N, S, S W, E, N, W”. As the array was drawn from anew for each block, block sequences within a session were extremely unlikely to be alike or even similar to one another and each session's order of stimulus presentation over the course of the 32 trials was unique. By using a pseudorandom sequence like this, no more than four consecutive trials could occur during which the up-gradient direction was associated with the same feeder. Thus, pigeons were not likely to develop a response bias toward any individual feeder.

**Figure 3:**
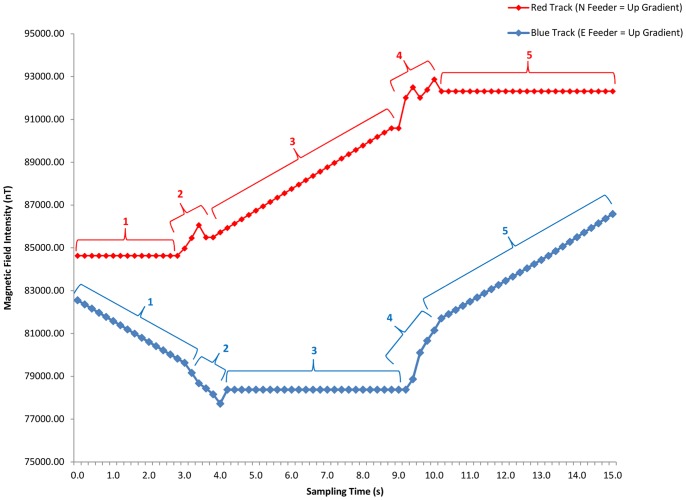
Magnetic field intensity experienced by a pigeon over the course of a trial's 15-s sampling period. Shown are intensities for red (left) and blue (right) track segments seen in Figure 2c.

Thus, as the pigeon moved around the periphery, the direction of the track in the VMI-map progressed according to the pigeon's position in the arena over time. If the pigeon sat still in one location, the track continued in the corresponding direction in the VMI-map until the pigeon changed location (see also Figures S1 for magnetic field parameters experienced by the pigeon when sitting still for 15 second sampling period in front of each feeder). Two feeders on one axis were associated with up and down movement along the magnetic intensity gradient (maximum rate of magnetic intensity change) and the other two feeders associated with sideway movement in the map (no magnetic intensity change). Thus, if a pigeon sat in front of the feeder-response unit associated with movement up the gradient, the track would move vertically upward on the map and the pigeon experienced the maximum rate of magnetic intensity increase (see Figure 2a&b). In contrast to this, sitting at one of the two feeder-response units associated with track movement sideways in the map along the map's lines of equal intensity left the current intensity value unchanged. Diagonal movements in the map, which occurred when the bird was located (usually while moving) between two feeders, resulted in intermediate rates of change in intensity depending on which feeder the bird was closest to and what the orientation of the gradient was in relation to the feeders for a given trial. To illustrate this, we plotted two 15-s example tracks (red and blue) within the VMI-map (Figure 2c) and related that to the pigeon's position in the arena (Figure 2d) as well as the magnetic field intensity experienced by the pigeon at its current position in the arena during the 15-s sampling period (Figure 3).

At the end of the 15 s sampling period, all four feeders' pecking lights were illuminated. The pigeon was required to indicate its choice by pecking one key. Three pigeons were trained to choose the feeder associated with up-gradient movement (increasing intensity) in the virtual map (up-gradient group) and three pigeons were required to select the downward-gradient (decreasing intensity) feeder (down-gradient group). A correct choice was rewarded with a 10-s access to the food magazine, whereas incorrect choices resulted in a time penalty of 10 s being added to the 5-s intertrial interval (ITI), during which the arena was dark and only the background magnetic field was present. Each conditioning session was terminated by four blocks of 8 trials having been completed (typically within 20 minutes) or a time limit (90 minutes) having been reached. In either case, if a pigeon made an incorrect choice during the last trial of the session, the same stimulus presentation of the last trial was repeated (not included in data analysis) until the bird made a correct choice to ensure that the session ended with the pigeon experiencing a positive reinforcement in connection with the correct stimulus. Following the pre-training described above, fifteen conditioning sessions were conducted with two birds from each group, but due to logistical constraints only 10 sessions were performed with the remaining two birds.

### Coil Control Procedures

For the Coils On-Off controls series, each of the four blocks of 8 trials that comprised a session was randomly divided into four Coils-On and four Coils-Off trials. During Coils-On trials the procedures described above where followed. By contrast, during Coils-Off trials the MVR software did not supply any current output from the three power supplies to the 3-axis magnetic coil system. Therefore, the relays in the control room were still producing audible clicks as if the direction of current coming from one or more of the power supplies were switched from clockwise to counterclockwise for a coil axis, but no magnetic field was produced by the coil system.

For the Parallel-Antiparallel control series, the number of each coil's wire loops was halved and a switch added that allowed the current in both halves of the coil to run either parallel (in the same direction) or anti-parallel (in opposite directions). Whilst the outer coil's 26 loops were halved into two sets of 13 loops, for the 11 loops of the inner coils, we added an additional loop of wire that was only supplied with current during the anti-parallel setting so that current ran through 6 loops in one direction and through 5+1 loop in the other direction. When running parallel, the same magnetic field intensity was produced as for standard sessions, but when running anti-parallel the two coil halves mostly cancelled each other out (Figures S2 and S4). A residual magnetic field intensity of between 1,300 and 3,700 nT (depending on the pigeon's position in the arena) with residual inclination and declination ranging between +0.8° to +3.0° and +0.5° to +2.5° respectively was still produced by the coils during the anti-parallel setting. This was probably due to the retro-fitting of the double coils system, which may not have exactly halved the coils.

### Statistical Analysis

**Figure 4:**
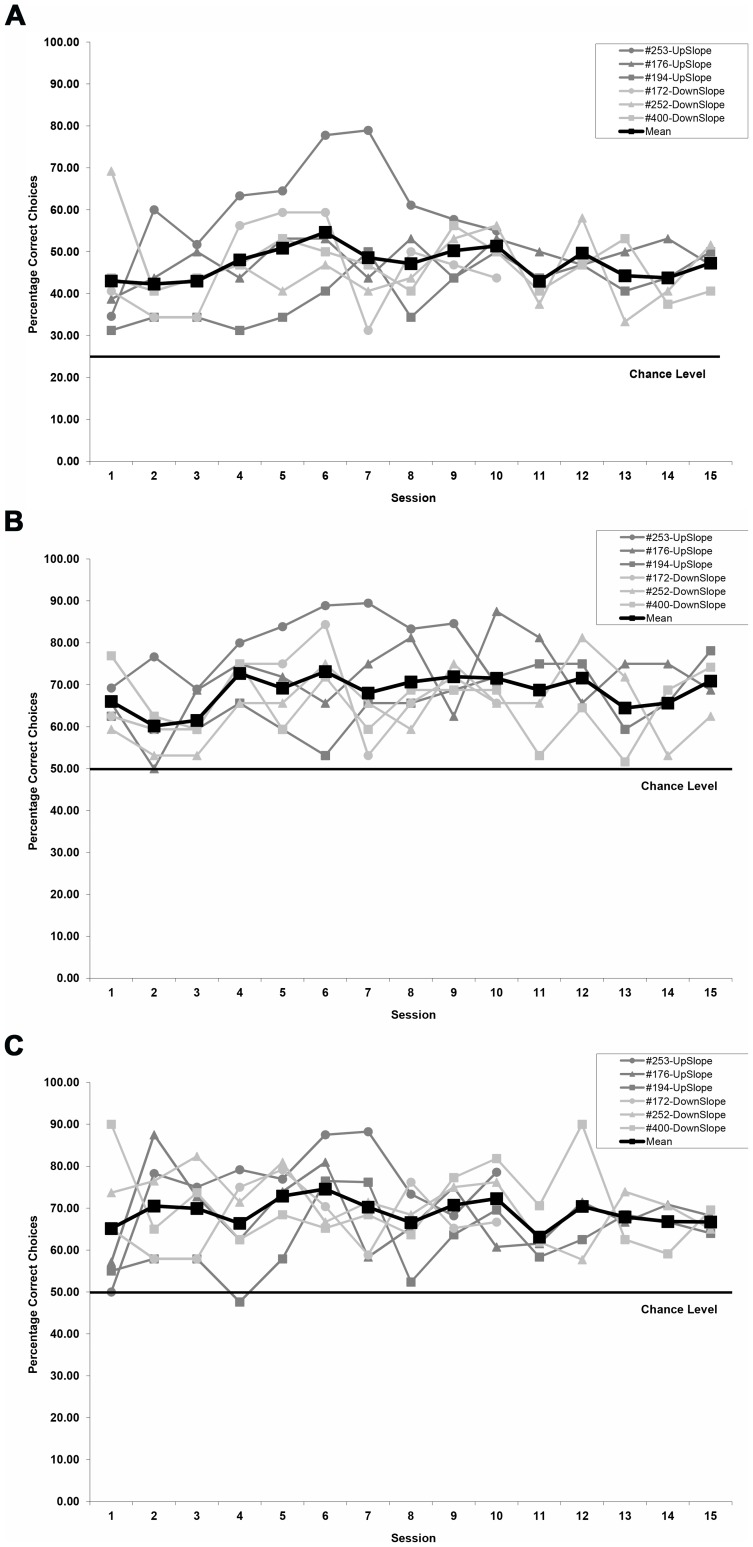
Mean percentage of correct choices made by individual pigeons during each session of the initial conditioning series. a) Mean percentage of correct choices for the two groups, for which rewarded feeder choices were either associated with increasing (up-gradient) or decreasing (down-gradient) intensity changes (chance level 25%). Because of extensive pre-training exposure to the reinforcement contingencies (see text), there was no evidence for gradual response acquisition typically associated with the learning of a discrimination task. b) Combined mean percentage of choices made at either feeder associated with the intensity gradient of the virtual magnetic intensity (VMI) map (note chance level of 50%). c) Mean percentage of correct choices calculated only for trials during which the pigeons chose either of the two feeders associated with the direction of the intensity gradient of the virtual magnetic intensity (VMI) map (chance level of 50%).

**Figure 5:**
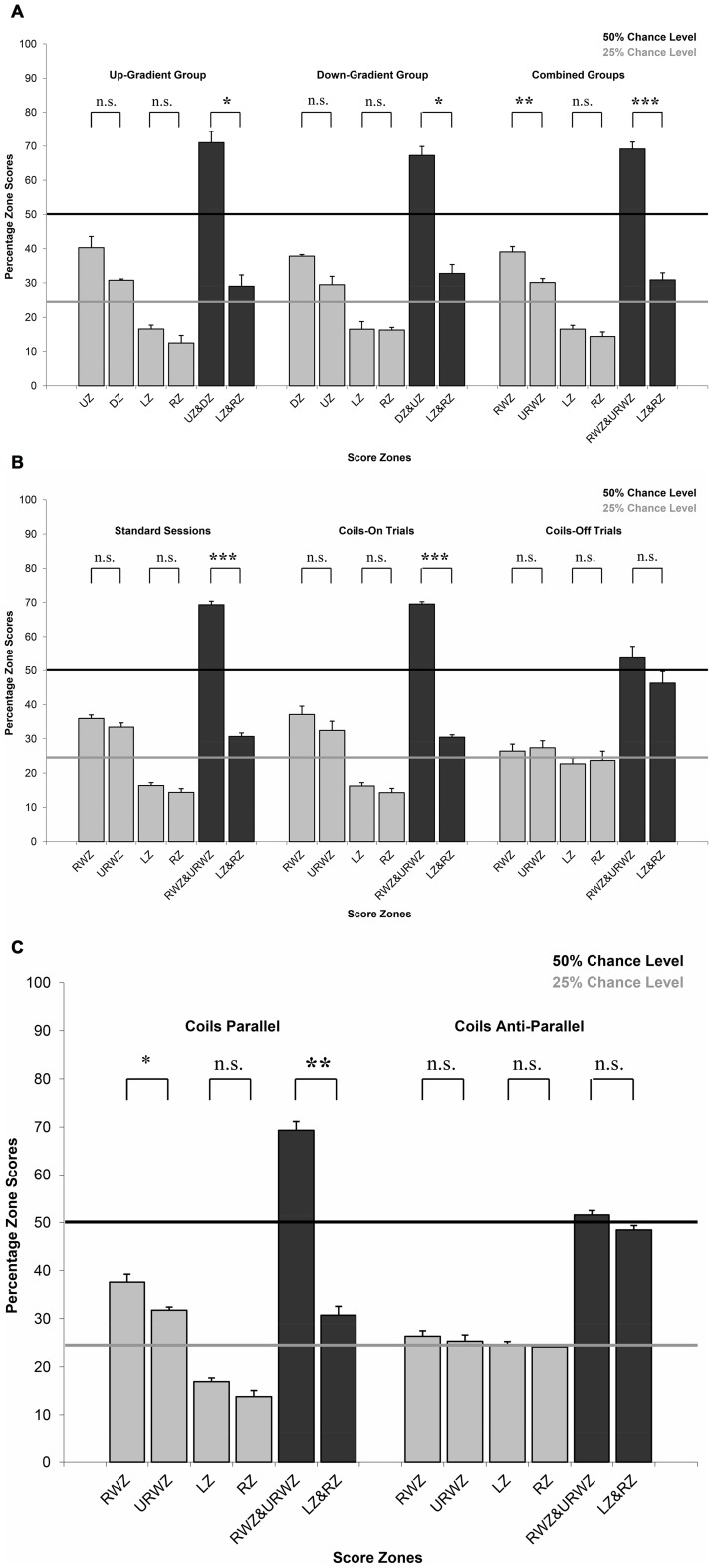
Mean percentage of zone scores with standard error bars showing in which VMI-map zone each trial's track terminated at the end of 15 s sampling period. a) Initial conditioning series for which pigeons were divided into groups as to whether feeder choices associated with increasing (up-gradient) or decreasing (down-gradient) intensity changes were rewarded (chance level 25%). b) Coils on-off series consisting of standard sessions as well as control sessions for which half of the trials had normal current input to the magnetic coil system and the other half had no current input based on a pseudorandom schedule. Non-significance does not imply random discrimination performance as this score relates to the amount of time spent at each feeder during the 15-second sampling period, which may be different from final feeder choice (see also Figure 2c). c) Parallel-anti-parallel series for which sessions with current running parallel through a double-wound coil system were alternated with sessions with current running anti-parallel. For abbreviations see Figure 4 except: UZ  =  Up gradient zone, DZ  =  Down gradient zone, LZ  =  Left gradient zone, RZ  =  Right gradient zone, RWZ  =  rewarded gradient zone (UZ or DZ for up-gradient and down-gradient groups respectively) and URWZ  =  unrewarded gradient zone (DZ or UZ for up-gradient and down-gradient groups respectively). Levels of significance: n.s.  =  not significant, *  = 0.05; **  = 0.01; and ***  = 0.001.

For each session performed by each bird, the percentage of correct choices out of 32 trials was calculated. We also calculated for each session the mean discrimination performance across all birds, which was graphed together with the individual birds' percentage of correct choices for each session (Figures 4 and 5). Next the mean percentage of correct choices across all sessions was calculated for each individual bird with standard error values based on the number of birds. These mean values for individual birds were our independent measures for all statistical tests except the non-parametric Wilcoxon Signed Ranks test (see below).

Because percentages ranging from 0 to 100% form a binomial rather than a normal distribution, with the deviation from normality being great for small or large percentages (0 to 30% and 70 to 100%; [37]), we performed an arcsine transformation for all percentage values prior to parametric statistical analyses. Groups of two means were compared using paired or un-paired two-sample T-tests and groups of more than two means with a one-way ANOVA. For the initial conditioning series we also fitted a Linear Mixed Model ANOVA to the data set using SPSS software 19 (SPSS Inc.) to examine the data for the occurrence of learning, detect any changes in behavior over time due to increased experience with the experimental setup and reinforcement contingencies, as well as to estimate any autocorrelations between sessions. Whether or not the discrimination performance was different from chance level was assessed using 95% confidence intervals, un-paired two-sample T-tests as well as the Wilcoxon Signed Ranks test, the latter testing whether discrimination performance was consistently different from chance level over the course of each experimental series (all statistical tests see [37]).

The tracks plotted by the MVR software in the VMI-map were scored for each session according to within which zone they terminated at the end of the 15-s sampling period (Figure 2b&c). For this purpose the map was divided into four score zones with each zone being defined as ±45° on either side of the slope axis (UZ  =  up zone and DZ  =  down zone) and of the two sideways directions (LZ – left zone, RZ – right zone) in the map with the pigeon's “release location” at its center. On the rare occasion (less than once per 500 trials) that a track terminated on the border between two zones, the zone that the majority of the pigeon's track was in was scored.

**Figure 6:**
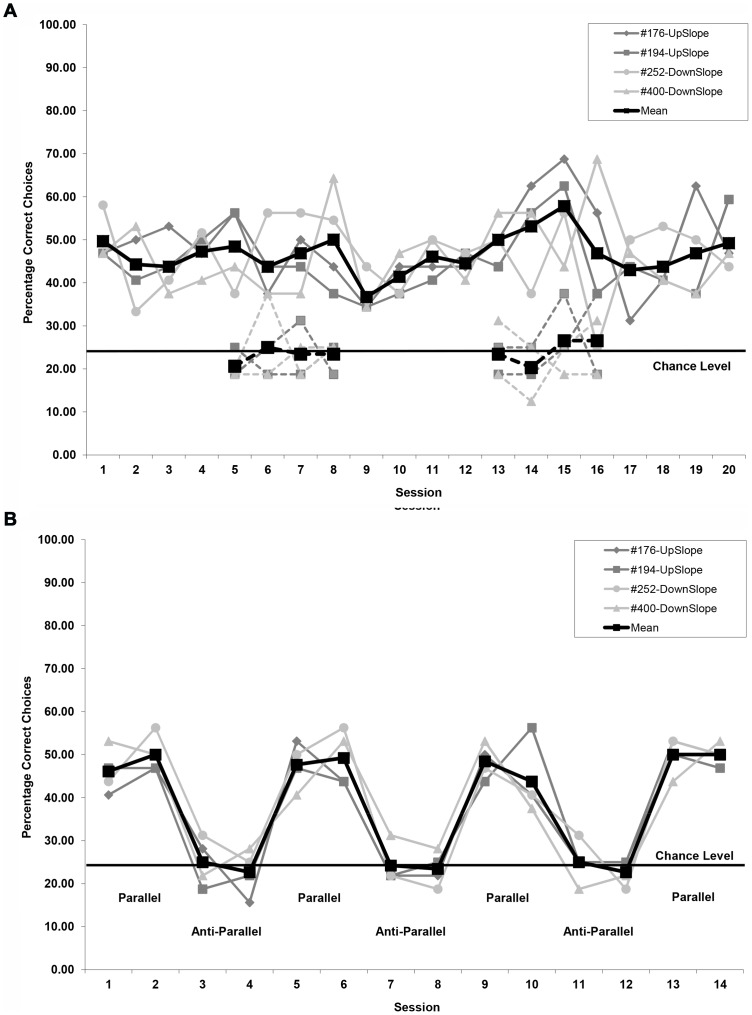
Mean percentage of correct choices made by individual pigeons during each control series. a) Coils on-off series consisting of standard sessions as well as control sessions for which half of the trials had normal current input to the magnetic coil system (solid symbols with solid lines) and the other half had no current input based on a pseudorandom schedule (solid symbols with dashes lines). b) Parallel-anti-parallel series for which sessions with current running parallel through a double-wound coil system were alternated with sessions with current running anti-parallel.

Similar to the percentage correct choice values, the mean percentage zone scores across all sessions were calculated for each individual bird with standard error values based on the number of birds and graphed (Figure 6). These mean values for individual birds were our independent measures for all statistical tests after performing an arcsine transformation (see above). Groups of two means were compared using paired or un-paired two-sample T-tests. Whether or not the zone scores were different from chance level was assessed using 95% confidence intervals as well as un-paired two-sample T-tests (all statistical tests see [37]).

### Ethics Statement

This study was carried out in strict accordance with the recommendations in the Guide for the Care and Use of Laboratory Animals of the National Institutes of Health. The protocol was approved by the Institutional Animal Care and Use Committee of Bowling Green State University (Permit Number: 09–001).

## Results

### Detection of Magnetic Intensity Change with Correct Directionality

Our results clearly show that homing pigeons are not only able to discriminate change in magnetic field intensity from no change or less rapid change in intensity, but even more interestingly, they are capable of differentiating the directionality of the intensity change as being either increasing or decreasing. Individual performance for the discrimination task, which required the pigeons to select the one feeder out of four available associated with intensity change in the correct direction, ranged between 31% and 79% correct choices for the 32-trial sessions (chance level 25%; Figure 4a). No statistically significant difference in the mean discrimination performance (unpaired T-test: T = 0.618; P>0.05) was detected between the up-gradient (n = 3, mean  = 49.77%±5.78 SE) and down-gradient (n = 3, mean  = 46.18%±0.36 SE) groups indicating the pigeons were equally well equipped to detect either direction of intensity change. We therefore grouped all six pigeons for further analysis with the mean performance across all birds ranging from 42% to 55% across sessions.

Furthermore, we did not observe any statistically significant change in the pigeons' performance over the course of the sessions (Linear Mixed Model ANOVA, type III test of fixed effects: F_Session_  = 2.920, p = 0.092), i.e., there was no traditional acquisition curve for the conditioned response. This was not unexpected as the pigeons were already exposed to the reinforcement contingencies during pre-training (see Methods and Materials).

As there was no change in performance over time, we calculated the mean performance over all sessions for each bird and then the mean discrimination performance across all birds (n = 6, mean 47.97%±2.71 SE, 95% confidence interval 41.00% to 54.95%). This was significantly different from chance level (25%), both when comparing individual mean bird performances to chance level (un-paired T-test: T = 8.849, P<0.001) as well as when looking at the birds' mean performance for each session over time (Wilcoxon Signed Ranks Test: T-Value  = 0, p<0.001), thus indicating that the pigeons were able to perform the discrimination task and that performance was consistently above chance level over time. A systematic difference between subjects was detected (Linear Mixed Model ANOVA, type III test of fixed effects: F_Subject_  = 7.908, p<0.001) due to bird 253′s higher performance level. We did not observe any within-session effect as there was no significant change in performance across the four 8-trial blocks, which composed individual sessions (1^st^ block: 44.22%±4.73 SE, 2^nd^ block: 50.16%±4.07 SE, 3^rd^ block: 48.78%±1.60 SE, 4^th^ block: 48.38%±2.70 SE; One-way ANOVA: F = 0.542, p = 0.659).

### Distribution of Errors

The distribution of erroneous choices was not random. When correct feeder choices were combined with those for the feeder opposite the correct one, that is, the feeder associated with movement along the gradient in the opposite direction from the one rewarded, then axial mean performance across birds ranged from 60% to 73% (chance level 50%; Figure 4b). Due to an increase in discrimination performance by bird 253 after session 3, there was a significant change in performance over the course of the sessions (Linear Mixed Model ANOVA, type III test of fixed effects: F_Session_  = 6.230, p = 0.015) and a significant difference between subjects (Linear Mixed Model ANOVA, type III test of fixed effects: F_Subject_  = 6.890, p<0.001). Mean performance over all sessions (n = 6, mean 68.98%±2.31 SE, 95% confidence interval 63.05% to 74.92%) was significantly different from chance level (50%), both when comparing individual mean bird performances to chance level (un-paired T-test: T = 7.562, P<0.001) as well as when looking at the birds' mean performance for each session over time (Wilcoxon Signed Ranks Test: T-Value  = 0, p<0.001).

This is particularly interesting as this means that almost half of all the incorrect choices (40.29%) were made at the feeder in the opposite gradient direction rather than the two feeders associated with sideways movement in the map. Thus, most errors made were only an error in identifying the directionality of magnetic intensity change rather than a failure to detect the spatial orientation of the gradient itself. Nevertheless, had the birds only identified the feeders associated with the intensity gradient and then made chance choices as to whether choosing the up-gradient or down-gradient feeder along this axis, then we would expect the percentage of choices observed in Figure 4a to have roughly doubled when choices along both directions of the axis were combined for Figure 4b. This was not the case, as doubling the lower 95% confidence limit for the overall mean discrimination performance for choosing the correct feeder (82.00%) was considerably above the upper 95% confidence limit (74.92%) for the mean combined number of choices along either direction of the gradient. This is further supported by the fact that the six pigeons' VMI-tracks significantly more often terminated in the RW zone than the URWZ zone along the gradient direction (Figure 5a and see text below) indicating that the pigeons spend more time at the rewarded feeder than the unrewarded feeder associated with the opposite direction of the gradient.

### Distribution of Choices Along Gradient Axis

Whilst the above results already indicated that the pigeons were not only able to detect the orientation of the gradient axis, but also were able to select the correct feeder associated with the food reward on this axis (up- or down-ward gradient direction), we performed an additional analysis to confirm that the pigeons were not achieving their discriminative performance along the gradient axis via an alternative strategy. This is because one possibility, albeit unlikely, was that the pigeons first discriminated successfully the two feeders associated with the gradient and but then performed only around chance level when selecting one of the feeders along the axis.

For this analysis we included for each session only trials for which the bird had made a feeder choice associated with either increasing or decreasing intensity changes. That is, for example, if for a given trial the upward gradient was associated with the North feeder, then a choice at the North or South feeder was included in the analysis, whilst a choice at the East or West feeder would have excluded this trial from the analysis. We then calculated the percentage of correct feeder choices made along the gradient axis.

The mean performance across birds ranged from 63% to 75% (chance level 50%; Figure 4c). Mean performance over all sessions (n = 6, mean 69.42%±1.83 SE, 95% confidence interval 64.72% to 74.12%) was significantly different from chance level (50%), both when comparing individual mean bird performances to chance level (un-paired T-test: T = 10.106, P<0.001) as well as when looking at the birds' mean performance for each session over time (Wilcoxon Signed Ranks Test: T-Value  = 0, p<0.001). Thus, the pigeons were clearly able to discriminate between the feeders associated with the increasing and decreasing magnetic intensity cues along the gradient axis.

### Zones Score Analysis

Finally, we analysed in which of four possible zones on the VMI-map (see Figures 2b & 2c) each pigeon's track ended in at the end of each trial's 15 s sampling period. Analysis of the map zone scores showed that for both the up-gradient and down-gradient groups the pigeons' tracks ended significantly more in the map sector associated with the rewarded gradient direction (UZ for up-gradient group: mean 40.27%±3.29 SE; DZ for down-gradient group: mean 37.81%±0.48 SE) and, secondarily, in the one associated with the unrewarded gradient direction (DZ for up-gradient group: mean 30.74%±0.38 SE; UZ for down-gradient group: mean 29.44%±2.47 SE) than either of the map sectors linked to sideways movement in the map (LZ for up- and down-gradient groups respectively: mean 16.55%±1.16 SE and 16.49%±2.26 SE; RZ for up- and down-gradient groups respectively: mean 12.44%±2.23 SE and 16.25%±0.79 SE) (Figure 5a). Due to the small number of birds in each group, only combined zone scores for the rewarded and unrewarded gradient direction were significantly greater than for the combined sideway zone scores (paired T-test: T = 5.811 and 6.325 for up-gradient and down-gradient groups respectively, both P<0.05). Therefore, based on simple trigonometry, for the tracks to have terminated predominantly in the UZ and DZ sectors, pigeons must have spent more than half of the 15-s sampling period in front of the UZ and DZ feeders, but this must have also predominantly occurred early on in a trial indicating that the pigeons were relatively quickly able to identify the feeders associated with the gradient.

As was described above for the percentage of correct choices, no significant difference was detected between the up-gradient and down-gradient groups for either the zone scores associated with the rewarded (unpaired T-test: T = 0.732, P>0.05) or unrewarded (unpaired T-test: T = 0.538, P>0.05) gradient direction. Thus, we combined the zone scores for both groups. Most importantly, the mean rewarded zone scores for the six birds averaged across all sessions was significantly different from chance (25%, n = 6, mean 39.04%±1.59 SE, 95% confidence interval 34.96% to 43.12%; un-paired T-test: T = 9.365, P<0.001). Also, the rewarded zone score was significantly greater than unrewarded zone score (paired T-test: T-values  = 5.363, p<0.01; Figure 5a). The combined rewarded and unrewarded zone scores averaged across all sessions for all six pigeons was significantly different from chance level (50%; n = 6, mean 69.13%±2.08 SE, 95% confidence interval 63.79% to 74.48%; un-paired T-test: T = 8.625, P<0.001) and significantly greater than the combined sideway zone scores (paired T-test: T-values  = 10.000, p<0.001), whereas there was no difference between the left and right zone scores (paired T-test: T-values  = 0.096, p>0.05). When the up-gradient and down-gradient groups were combined, it was thus evident that not only did the pigeons determine within less than 7 seconds which two feeders were associated with the intensity gradient (otherwise the tracks could not have terminated predominantly in the UZ and DZ sectors as described above), but furthermore, they were able to discern which of these two was the feeder associated with the right directionality in intensity change.

### Coil On-Off Control Sessions

White noise was used to mask any humming sounds emanating from the coils and the gradient direction was disassociated from any visual cues in the experimental room by selecting on a pseudorandom schedule which of the four feeders in the arena was associated with up-gradient movement in a given trial. To test whether any other alternative cues may have been used by the pigeons to identify the correct feeder, we conducted a coils on-off series of control sessions with four of the original six pigeons. Two sets of four control sessions (half of the trials Coils-On and half of the trials Coils-Off in pseudorandom order) were alternated with four consecutive standard sessions (same procedure as for the initial conditioning series). Mean discrimination performance of the feeder associated with the rewarded gradient direction averaged across all birds was 44.71%±0.75 SE (n = 4, 95% confidence interval 42.33% to 47.10%) for all standard sessions and 49.61%±1.49 SE (n = 4, 95% confidence interval 44.86% to 54.37%) for the Coils-On trials of the control sessions (Figure 6a). This was in both cases significantly above chance level (25%; un-paired T-test: T = 27.736 and 17.246, both P<0.001). The birds' mean performance for each session was in both cases also consistently above 25% over time (Wilcoxon Signed Ranks Test: both T-Value  = 0, respectively p<0.001 and p<0.01). The performance in standard sessions and for the Coils-on trials was in strong contrast (One-way ANOVA: F = 203.660, p<0.001) to the performance for the Coils-Off trials (n = 4, mean 23.67%±0.49 SE, 95% confidence interval 22.12% to 25.22%), during which performance fell to around chance level (25%; un-paired T-test: T = 2.415, P>0.05; Wilcoxon Signed Ranks Test: T-Value  = 9, p>0.05). Therefore, the pigeons were clearly not able to perform the discrimination task when the coils were turned off.

As observed during the initial conditioning series, the combined rewarded and unrewarded gradient zone score averaged across all four pigeons for the standard sessions (mean 69.34%±1.05 SE) and Coils-On trials (mean 69.53%±0.71 SE) was in each case significantly greater than the respective combined sideways zone score (mean 30.66%±1.05 SE and mean 30.47%±0.71 SE; paired T-test: T-values  = 17.3907 and 25.9031 respectively, both p<0.001; Figure 5b). Importantly this was in strong contrast to the combined rewarded and unrewarded gradient zone score for the Coils-Off trials (mean 53.71%±3.41 SE; One-way ANOVA: F = 19.065, p = 0.001) and it is significant that there was no difference in the combined zone scores for the gradient zones versus the sideways zone for the Coils-Off trials (paired T-test: T-value  = 1.0880, p>0.05).

The non-significant differences between the rewarded and unrewarded gradient zone scores for standard sessions and Coils-On trials (paired T-test: T-values  = 1.2125 and 0.9197, both p>0.05) were no reflection of the birds being unable to discriminate the intensity cues in this experiment. Instead they were caused by the birds choosing to spend an equal amount of time especially during the early part of the sampling period at the two feeders associated with up-gradient or down-gradient intensity change thus resulting, based on simple trigonometry, in most zone scores falling into these two zones (see Figure 2c). Final feeder selection nevertheless significantly favoured the rewarded feeder of these two as had been observed during the initial conditioning series (see Figure 6a). Thus, when magnetic cues are only intermittently available within individual sessions, the birds seemed to focus their attention more on the detection of the gradient orientation rather than the directionality of the intensity change.

### Coils Parallel-Anti-Parallel Control Sessions

To eliminate any other alternative cues (e.g., heat or vibration) potentially associated with the varying amounts of current passing through the coils during a trial we next conducted a parallel-antiparallel control series using a double-wrapped coils as suggested by [38]. Four sets of two sessions with the current running through the double-wound coils in the same direction (parallel sessions, i.e., same magnetic intensity cues as for the initial conditioning series) were alternated with three sets of two sessions with the current running in opposite direction (anti-parallel sessions, i.e., background magnetic intensity cues) (Figure 6b). Similar to the Coils On-Off control experiment, for parallel coils sessions the mean discrimination performance of the feeder associated with the rewarded gradient direction averaged across all birds (n = 4, mean 48.14%±0.51 SE, 95% confidence interval 46.51% to 49.78%) was significantly above chance level (25%; un-paired T-test: T = 47.329, P<0.001; Wilcoxon Signed Ranks Test: T-Value  = 0, p<0.01). This was significantly different (paired T-test: T-value  = 9.7269, p<0.01) from the mean performance for each bird for the anti-parallel coils sessions (n = 4, mean 23.83%±0.54 SE, 95% confidence interval 22.12% to 25.54%; chance level 25%; un-paired T-test: T = 2.178, P>0.05; Wilcoxon Signed Ranks Test: T-Value  = 3, p>0.05). This clearly shows that the pigeons were not able to perform the discrimination task when the current ran anti-parallel through the coils.

Analysis of the zone scores showed the same overall pattern observed for the Coils On-Off control experiment (Figure 5c). The combined rewarded and unrewarded gradient zone score for the parallel sessions (n = 4, mean 69.31%±1.86 SE) was significantly greater than the combined sideways zone score (n = 4, mean 30.69%±1.86 SE; paired T-test: T-value  = 9.8837, p<0.01) as well as in strong contrast to the combined gradient zone score for the anti-parallel sessions (n = 4, mean 51.56%±0.93 SE; paired T-test: T-value  = 9.7269, p<0.01). There was also a significant difference between the rewarded and unrewarded gradient zone scores for parallel sessions as had been observed for the initial training series (paired T-test: T-value  = 3.3727, p<0.05), but not for anti-parallel sessions (paired T-test: T-value  = 0.4595, p>0.05). The birds were therefore spending most of their time and early on during the sampling period in front of the rewarded feeder during parallel but not during anti-parallel sessions.

Discrimination performance during both Coils On-Off and the Parallel-Antiparallel series fell to just below chance level with relatively little variance. The birds were still very motivated during coils-off trials as well as during anti-parallel sessions to move between feeders and peck the response keys when they were lit, i.e., they did not make their choices completely randomly nor did they just sit in front of a single feeder for the entire session pecking only that response key. Instead they adopted a combination of alternative choice behaviours, with the combination being unique to each bird. Examples of such behaviours were “win-stay-loose-shift” (the bird stayed at a feeder that during the last trial had produced a reward until this was no longer the case, at which point it moved to the next feeder) or moving clockwise or counter-clockwise from feeder to feeder either every trial or every few trials producing quite a consistent mean performance of just below 50%.

As described above, retrofitting our coil system for the anti-parallel sessions resulted in a weak residual magnetic intensity gradient being produced by the coils instead of complete cancellation of the coils' fields, yet the pigeons' discrimination performance fell to chance level during anti-parallel sessions. This is not surprising as such a weak stimulus would be considerably more difficult to discriminate and thus the birds were highly likely to switch for the same level of motivation (85% free-feeding weight and 10s feeder access per correct choice) to alternative behavioural strategies (see above), which still yielded a reward for 50% of the trials. This is especially true given that the birds were only exposed to this weaker stimulus for two sessions at a time and for a total of only 6 sessions. Therefore, no conclusions can be drawn from this control experiment about whether not the pigeon are able to perceive such shallow magnetic intensity gradients. Instead a carefully designed threshold study will need to be performed in the future.

In summary, because discrimination performance fell to chance level not only when current to the coils was disconnected, but also when current ran through the coil system in an anti-parallel fashion, the two control experiments demonstrated that neither the current itself nor any other alternate non-magnetic cues could have been used by the birds to discriminate the magnetic intensity cues in this experimental setup. This result is consistent with the fact that the coils felt barely warm to touch during sessions, the arena's support base rested on a concrete floor without contact to the coils, and auditory as well as visual cues were controlled for.

## Discussion

Similar to the classical sun compass experiments by Kramer [39] and Hasler *et al.* [40], in which they conditioned starlings and fish, respectively, in an orientation arena to the position of the sun, the behavioural paradigm presented here combined conditioning procedures with the traditional orientation arena technique, the latter of which had been previously used for measuring spontaneous responses in sea turtles in the presence of specific magnetic field values (e.g., [35]). For this new approach, the pigeons were required to use magnetic intensity cues to solve a spatial orientation task in the absence of all other sensory cues, and as such, this went beyond the traditional discrimination of the presence versus absence of a magnetic field anomaly tested in previous magnetic conditioning studies (e.g., [26]–[29]).

The pigeons clearly were able to discriminate between the two feeders associated with the maximum rate of change in magnetic field intensity and the two feeders associated with no or a slow rate of change in intensity (very slow change rather than no change commonly occurred as the tracker arm was often not perfectly aligned with the feeder due to slight variations in the pigeon's position in front of the feeder). Furthermore, out of the two feeders associated with the intensity gradient, the pigeons chose significantly more often the rewarded directionality in magnetic intensity change.

Any changes in inclination and/or declination that occurred in conjunction with the intensity changes experienced by the pigeon (Figures S1 & S2) were of the same magnitude as the NW-SE gradient in background inclination (i.e., at a 45° angle to the axis of background variation) as well as the more complex variation in declination across the experimental arena caused by the structural steel and electrical circuits in the walls of the experimental room (Figures S5 & S6). These variations in inclination and declination thus produced spatially and temporally very complex and extremely weak inclination and/or declination signals without any clear relationship as to which was the correct feeder.

Newts have been shown to be highly sensitive to inclination changes, potentially to within 1/10^th^ of a degree (e.g., [6]). In our study, inclination and declination changes at the individual feeders were up to 1/3 of a degree and 1° respectively over 15 seconds (Figures S1 & S3). Whilst the same level of sensitivity as in newts has not yet been demonstrated in homing pigeons, we nevertheless need to consider that the pigeons could have used such small changes to assist their discrimination performance, especially when sitting for extended periods at the South feeder (and to a lesser extent at the West feeder) where intensity changes were correlated with the greatest inclination changes. Furthermore, it has been suggested that if the avian magnetic compass is located in the retina of the eye and the birds “see” magnetic field direction as a visual pattern projected on the retina, then different visual patterns may be associated with different levels of intensity [41]. It could therefore theoretically be possible that the pigeons in this study would have successfully discriminated the magnetic cues by comparing the visual patterns associated with either increasing or decreasing magnetic intensity.

There are several reasons, however, why discrimination based on these small inclination changes is very unlikely. Firstly, no preference for the South (or West) feeder was observed. Secondly, to experience these small inclination or declination changes in a consistent pattern, the pigeons would have had to sit very still with their head in almost exactly the same position at the feeder for up to 15 seconds, or otherwise the spatial variations in the background field throughout the arena (see above) would have masked these small changes. The pigeons typically did not remain still but rather moved side to side by up to 20 degrees on either side of the feeder when positioning themselves at the feeders. Finally, it is at this point highly speculative as to whether pigeons could perceive such rapidly changing patterns on the retina or even whether such rapidly changing patterns would be even produced on the retina.

In contrast to the above described changes in inclination and declination, the intensity changes experienced by the pigeons were very strong as well as spatially and temporally consistent as a discriminative cue. Therefore, we conclude that the discrimination performance we observed was most likely based on intensity perception and that the pigeons were able to distinguish between increasing versus decreasing magnetic field intensity as associated with upward and downward movement along the map's gradient direction.

Whilst it had been previously demonstrated that pigeons are able to discriminate the presence and absence of a magnetic anomaly varying mostly in intensity (peak intensity of 189 μT compared to 44 μT background intensity), but nevertheless varying also significantly in inclination (peak inclination of –80° compared to –64°) [29], the results presented here show for the first time that pigeons are able to detect magnetic intensity as a salient cue by itself and can discriminate changes in intensity and even changes in the direction of intensity change to solve a spatial orientation task. Furthermore, visual inspection of the tracks recorded in the VIM-map showed the pigeon frequently entering and remaining in either the up- or down-gradient zone already early during the sampling phase. This observation was confirmed by the zone score results with significantly more tracks terminating in these two zones. Thus, the pigeons were able to determine either the correct feeder or the feeder axially opposite the correct one within only a few seconds of sampling time and positioned themselves in front of either of these feeders for most of the sampling phase.

Furthermore, the pigeons' sensitivity level was significantly greater than anything previously shown for homing pigeons as past magnetic conditioning experiments [27]–[34] always involved magnetic anomalies with peaks several times Earth strength. Whenever the pigeon's tracker arm was perfectly aligned with the feeder position, and thus the pigeon's track in the VMI- map moved straight up or down the map's gradient, the rate of change was 833 nT per second. Thus, the pigeon experienced a maximum change of 12,500 nT during the 15 s sampling period (approximately ¼ of the local Earth background intensity). Typically such perfect alignment of the tracker arm with the feeder position was not achieved and/or the pigeon did not sit completely still in front of the feeder, so that the pigeon experienced slower changes in intensity per unit time (625 nT/s for a tracker arm position 22.5° to the left or right of the center of the feeder, i.e., which is equivalent to a latitudinal movement of approximately 208 km/s on the Earth's surface). Pigeons also frequently selected their final feeder choice within only a few seconds, therefore reducing the absolute change in intensity (but not the rate or direction of change in intensity) experienced before identifying its feeder of choice. So while the sensitivity level demonstrated here is still insufficient for a theoretical magnetic map based on magnetic intensity, it is considerably closer to the sensitivity level of tens of nT to a few hundred nT required for such a map than has ever been experimentally demonstrated before with a conditioning task in a vertebrate species.

The discrimination performances observed for previous successful magnetic conditioning studies was typically significantly lower than the performances observed for discrimination tasks involving other sensory modalities (e.g., 80–95% for visual or auditory cues). Such past studies all involved 1) a spatially variable magnetic stimulus and 2) a behavioural response requiring movement by the animal, which are two prerequisites that appear to be necessary for successful magnetic conditioning to occur [42], although there is one notable recent exception using European robins (*Erithacus rubecula*) where discrimination of a magnetic anomaly was not achieved despite fulfilling the two criteria listed above [43]. Therefore stimulus, response and reinforcement were separated in space and time, making such magnetic discrimination tasks potentially more difficult for the animal resulting in lower performance. The results reported here show considerably higher discrimination (45 to 55% with 25% chance level. i.e., 20 to 30% above chance level) performance than observed in past successful magnetic conditioning studies (typically 60 to 70% with 50% chance level, i.e., 10 to 20% above chance level). This might be because the response behaviour was for the first time tied to a spatial orientation task putting the behavioural response thus into a more “natural” context, but further studies will be required to confirm this. More specifically, since pigeons have been observed in the field to follow magnetic field intensity contour lines or fly perpendicular to these lines (i.e., along the magnetic gradient direction) at least in some locations [15]–[16], a conditioning task requiring the identification of the direction of a magnetic field intensity gradient thus might have more closely simulated orientation behaviour previously observed in the field than in past studies.

The VMI-map approach used here is of course at this point only a methodological construct that allows us to simulate to the birds on a temporal scale magnetic intensity changes that they would normally experience at a spatial scale not reproducible for a pigeon flying or even walking in a magnetically controlled laboratory environment. That is, we attempted to transform the spatial variation in magnetic field intensity experienced in the field into a temporal one in the more confined space of a laboratory to study whether and how magnetic field intensity changes could be used by pigeons to solve spatial orientation tasks. The pigeons in our study are of course not “aware” that they are moving through a virtual map generated by a computer and what this map looks like, but rather are likely to respond directly to the spatial relationship of intensity changes experienced. Nevertheless, the results presented here represent a first step in understanding a candidate magnetic map for long-distance navigation by providing for the first time information as to what magnetic cues are available to a pigeon at the sensory level for a proposed magnetic map. The ability to detect magnetic intensity changes and identify the direction of the steepest magnetic intensity gradient (or conversely the direction of the magnetic intensity contour lines) has been long hypothesized to be a key requirement for the determining latitude and longitude, respectively, during the “map”-step of navigation [13]–[14], [44]–[45]. Therefore, as the results reported here demonstrate a behavioural ability to identify the rapid changes in magnetic intensity as well as the direction of such changes, one of the major theoretical requirements for magnetic map navigation is fulfilled.

Nevertheless, it will of course be critical to develop this paradigm further in such a way that we will be able to test in the future more directly whether or not the pigeons' ability to detect temporal variations in magnetic field intensity, as demonstrated here, is actually linked to usage in terms of spatial perception during homing. This is because there is the theoretical possibility, which of course is not necessarily mutually exclusive to usage during spatial perception, that pigeons utilize their sensitivity to temporal variations in magnetic intensity to detect daily variations in the Earth's magnetic field for use as a “zeitgeber” for circadian rhythms. We consider this possibility, however, to be very remote given that the rate of change in intensity during a 30 minute noon window would be ≤0.03 nT per second (if assuming an intensity change of 50 nT during this time frame) and the smallest estimates of sensitivity to change in intensity have been 1–10 nT based on frosted lenses experiments with homing pigeons [46] and the 25 nT threshold measurement for honey bees [47].

Furthermore, we would like to acknowledge that this new paradigm by itself is unlikely to solve all questions regarding a potential magnetic map in homing pigeons. This is because the homing behaviour of pigeons in the field is far more dynamic and individualistic in how the pigeon samples and perceives the environment than can by simulated in the laboratory environment. But if it can be demonstrated that the birds can learn a behavioural sequence to “return” to a simulated home location in the VMI-map, and even can learn different sequences for different “release sites”, then this will provide some insight into what homing pigeons are capable of. Such insight can then lead to more advanced field studies, which may circumvent some of the inherent difficulties with field studies described above by asking more targeted questions relating to the role of the Earth's magnetic field during position determination rather than asking only whether magnetic intensity cues are used at all as it had been the case in previous studies (e.g., [11], [49]–[50], [12], [48]). Therefore, this new paradigm and field studies are complementary approaches, each with their own set of advantages and limitations.

Finally, researchers have been looking for a candidate magnetic intensity receptor in homing pigeons and migratory birds for several decades. The difficulty lies with the fact that magnetic fields permeate tissue and thus do not require a large nor topographically organized sense organ, such as an eye, to focus the stimulus onto the receptor, thus making the search for magnetoreceptors potentially akin to a search for a needle in a haystack. While some progress has been made in relation to a putative receptor system for the magnetic compass in the retina [2], the discovery of an avian magnetoreceptor for intensity perception seems, however, as elusive as ever. We suggest that it is imperative to have a robust, spatially relevant behavioural paradigm, such as the one demonstrated here, before any real progress can be made in understanding the sensory/neural mechanisms underlying magnetic intensity. Furthermore, as mentioned above, such a paradigm can elucidate what magnetic information is perceived by the pigeon and thus may be available for use during map navigation. This is especially true after several recent publications not only called into question the existence of the hitherto most likely presumed candidate magnetic intensity receptor [25], namely an iron-mineral base structure in the pigeon's upper beak, but also suggested the pigeon's lagena in the inner ear an alternative possible location for a receptor [51], beside the eye and the beak.

We therefore suggest that the potential for this new approach is two-fold. Firstly, it has great potential, especially in conjunction with more advanced field studies, for studying how pigeons as well as various migratory species could use magnetic intensity cues during position determination. Two logical possibilities for advancing this paradigm are to make the orientation task required of the pigeon within the map more complex as well as to make the VMI-map itself more complex and thus more realistic. In case of the former, a pigeon could be “released” at different locations along the VMI-map's intensity gradient. The pigeon would then have to decide whether to move up or down the intensity gradient based on its position at the time of release and indicate through a behavioural response such as key pecking when it has reached a target zone along the gradient.

Secondly, this new behavioural paradigm now opens up the possibility for systematic and detailed studies of how magnetic intensity is perceived. Previous work with rainbow trout (*Oncorhynchus mykiss)* [26] has already suggested the potential involvement of iron-based magnetoreceptors in the olfactory epithelium as an alternative to receptors located in the upper beak, an area not yet investigated by [25]. Also, it will be important to further investigate the role the ophthalmic branch of the trigeminal nerve plays in carrying magnetic information to the brain, as suggested by previous studies with homing pigeons [29] and migratory birds [52]–[53], as well as which areas in the brain process such information with some earlier studies having shown hippocampal responses to magnetic field intensity stimuli [54], [48].

## Acknowledgments

We sincerely thank Russell Mora for the development of the MVR software, Andrew Wickiser for the construction of the experimental setup, as well as Tom Barnhardt and Tom van Handel for their generous supply of thorough-bred racing pigeons. We greatly appreciated the invaluable input by Shannon Thompson, Jean Adelphi-Long, and Susan Orosz during the development of the pigeon harness as well as the constructive comments regarding the experimental setup by Henrik Mouritsen. We sincerely thank Henrik Mouritsen, Michael Walker, John Phillips and one anonymous reviewer for their assistance with improving this manuscript. Shannon Thompson, Jean Adelphi-Long, and Merissa Acerbi were also involved with the pre-training of the pigeons. The assistance by Matt Cannon and Susan Orosz to ensure the general welfare of the pigeons was also greatly appreciated.

## Supporting Information

Figure S1 **Magnetic field measurements during magnetic coils parallel sessions.** Magnetic field intensity (top row), magnetic field inclination (middle row), and magnetic declination (bottom row) experienced by pigeon sitting during 15 second sampling period in front of North feeder (first column), East feeder (second column), South feeder (third column), and West feeder (fourth column) for trials with the magnetic intensity gradient of the VMI-map being associated with either the North feeder (red), East feeder (blue), South feeder (red), or West feeder (purple).
(TIF)

Figure S2 **Magnetic field measurements during magnetic coils anti-parallel sessions.** Magnetic field intensity (top row), magnetic field inclination (middle row), and magnetic declination (bottom row) experienced by pigeon sitting during 15 second sampling period in front of North feeder (first column), East feeder (second column), South feeder (third column), and West feeder (fourth column) for trials with the magnetic intensity gradient of the VMI-map being associated with either the North feeder (red), East feeder (blue), South feeder (red), or West feeder (purple).
(TIF)

Figure S3 **Magnetic field inclination measurements during magnetic coils parallel sessions.** Magnetic field inclination experienced by pigeon sitting during 15 second sampling period in front of a) North feeder, b) East feeder, c) South feeder, and d) West feeder (fourth column) for trials with the magnetic intensity gradient of the VMI-map being associated with either the North feeder (red), East feeder (blue), South feeder (red), or West feeder (purple). Please note y-axis scale has been adjusted for each graph to show any inclination changes within 1/10^th^ of a degree.
(TIF)

Figure S4 **Background magnetic field intensity and magnetic field intensity generated by the coil system measured throughout experimental arena.** The background field and the magnetic field parameters generated by the coil system were characterized with a FVM handheld 3-axis vector fluxgate magnetometer (Meda Inc.) at the head height of a walking pigeon at 25 points distributed throughout the experimental arena (center of arena, eight points at a distance of 15 cm from the center of the arena around the periphery of the arena at 45° intervals, 16 points at a distance of 30 cm from the center of the arena around the periphery of the arena at 22.5° intervals). Data points were then extrapolated and plotted as a meshgrid with the Splot function in GnuPlot 4.2 (patch level 3). The x- and y-axes show the location within the arena, with the center coordinate (0,0) being located at the center of the arena, and coordinates of 1.0 an 2.0 being representing 15 and 30 cm from the center of the arena respectively. The z-axis indicates magnetic field intensity. Measurements were made with the coils set to parallel (left column) or anti-parallel (right column) current flow with no current send to the coils (background field; top row) or with the VMI-software set either at the intensity gradient level for the trial start setting -15,000 nT (second row), the trial start setting (ca. 85,000 nT; third row), or the intensity gradient level for the trial start setting +15,000 nT (fourth row).
(TIF)

Figure S5 **Background magnetic field inclination and magnetic field inclination generated by the coil system measured throughout experimental arena.** The background field and the magnetic field parameters generated by the coil system were characterized with a FVM handheld 3-axis vector fluxgate magnetometer (Meda Inc.) at the head height of a walking pigeon at 25 points distributed throughout the experimental arena (center of arena, eight points at a distance of 15 cm from the center of the arena around the periphery of the arena at 45° intervals, 16 points at a distance of 30 cm from the center of the arena around the periphery of the arena at 22.5° intervals). Data points were then extrapolated and plotted as a meshgrid with the Splot function in GnuPlot 4.2 (patch level 3). The x- and y-axes show the location within the arena, with the center coordinate (0,0) being located at the center of the arena, and coordinates of 1.0 an 2.0 being representing 15 and 30 cm from the center of the arena respectively. The z-axis indicates magnetic field inclination. Measurements were made with the coils set to parallel (left column) or anti-parallel (right column) current flow with no current send to the coils (background field; top row) or with the VMI-software set either at the intensity gradient level for the trial start setting -15,000 nT (second row), the trial start setting (ca. 85,000 nT; third row), or the intensity gradient level for the trial start setting +15,000 nT (fourth row).
(TIF)

Figure S6 **Background magnetic field declination and magnetic field declination generated by the coil system measured throughout experimental arena.** The background field and the magnetic field parameters generated by the coil system were characterized with a FVM handheld 3-axis vector fluxgate magnetometer (Meda Inc.) at the head height of a walking pigeon at 25 points distributed throughout the experimental arena (center of arena, eight points at a distance of 15 cm from the center of the arena around the periphery of the arena at 45° intervals, 16 points at a distance of 30 cm from the center of the arena around the periphery of the arena at 22.5° intervals). Data points were then extrapolated and plotted as a meshgrid with the Splot function in GnuPlot 4.2 (patch level 3). The x- and y-axes show the location within the arena, with the center coordinate (0,0) being located at the center of the arena, and coordinates of 1.0 an 2.0 being representing 15 and 30 cm from the center of the arena respectively. The z-axis indicates magnetic field declination. Measurements were made with the coils set to parallel (left column) or anti-parallel (right column) current flow with no current send to the coils (background field; top row) or with the VMI-software set either at the intensity gradient level for the trial start setting −15,000 nT (second row), the trial start setting (ca. 85,000 nT; third row), or the intensity gradient level for the trial start setting +15,000 nT (fourth row).
(TIF)
