# Supplementary figures and images for: Detection of Magnetic Field Intensity Gradient by Homing Pigeons (Columba livia) in a Novel “Virtual Magnetic Map” Conditioning Paradigm
Source: PLoS One. 2013 Sep 9;8(9):e72869. doi: 10.1371/journal.pone.0072869 (PMC3767695; doi:10.1371/journal.pone.0072869)

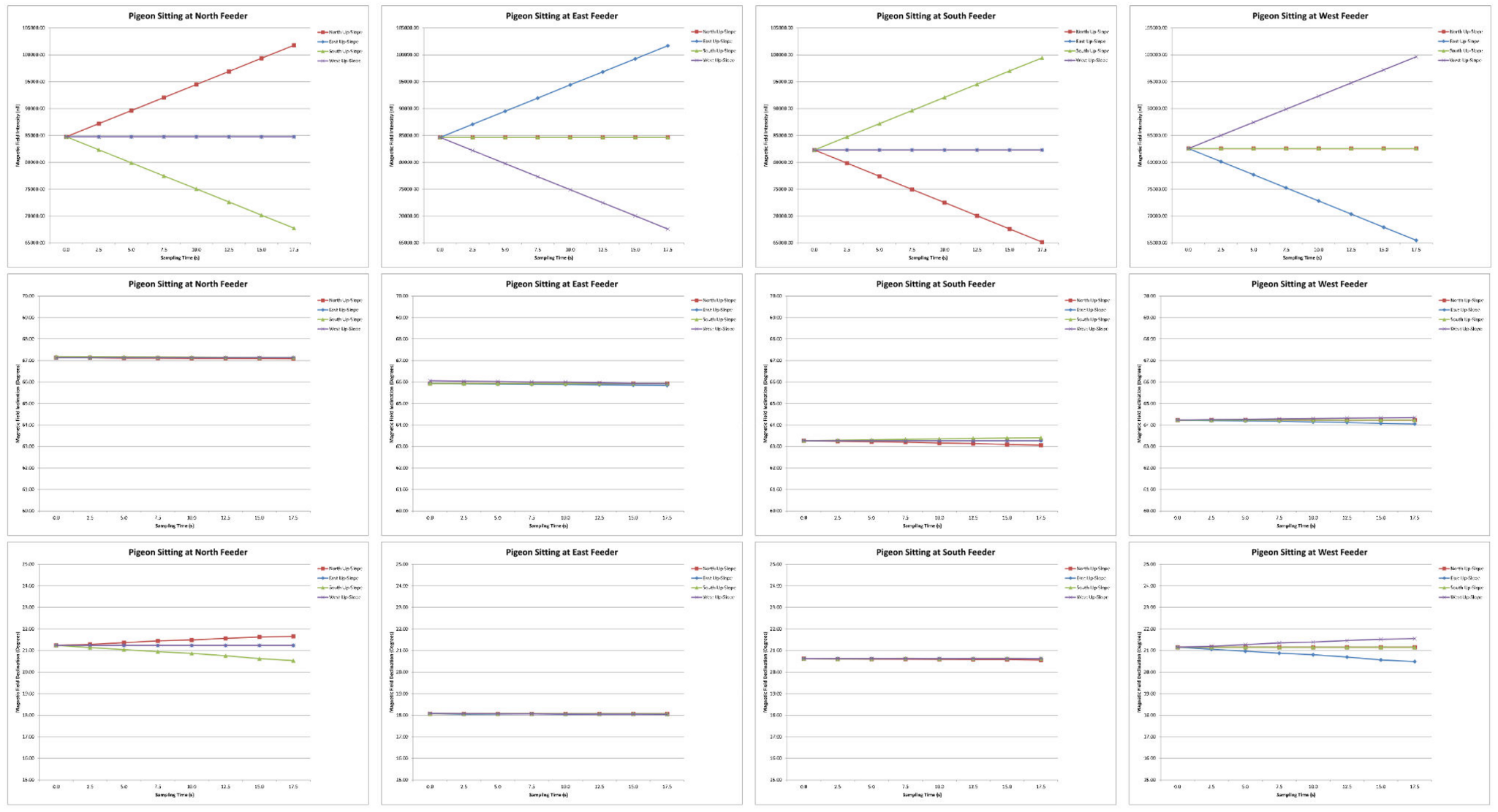

Supplement: Figure S1 — Magnetic field measurements during magnetic coils parallel sessions. Magnetic field intensity (top row), magnetic field inclination (middle row), and magnetic declination (bottom row) experienced by pigeon sitting during 15 second sampling period in front of North feeder (first column), East feeder (second column), South feeder (third column), and West feeder (fourth column) for trials with the magnetic intensity gradient of the VMI-map being associated with either the North feeder (red), East feeder (blue), South feeder (red), or West feeder (purple). (TIF) [file pone.0072869.s001.tif]

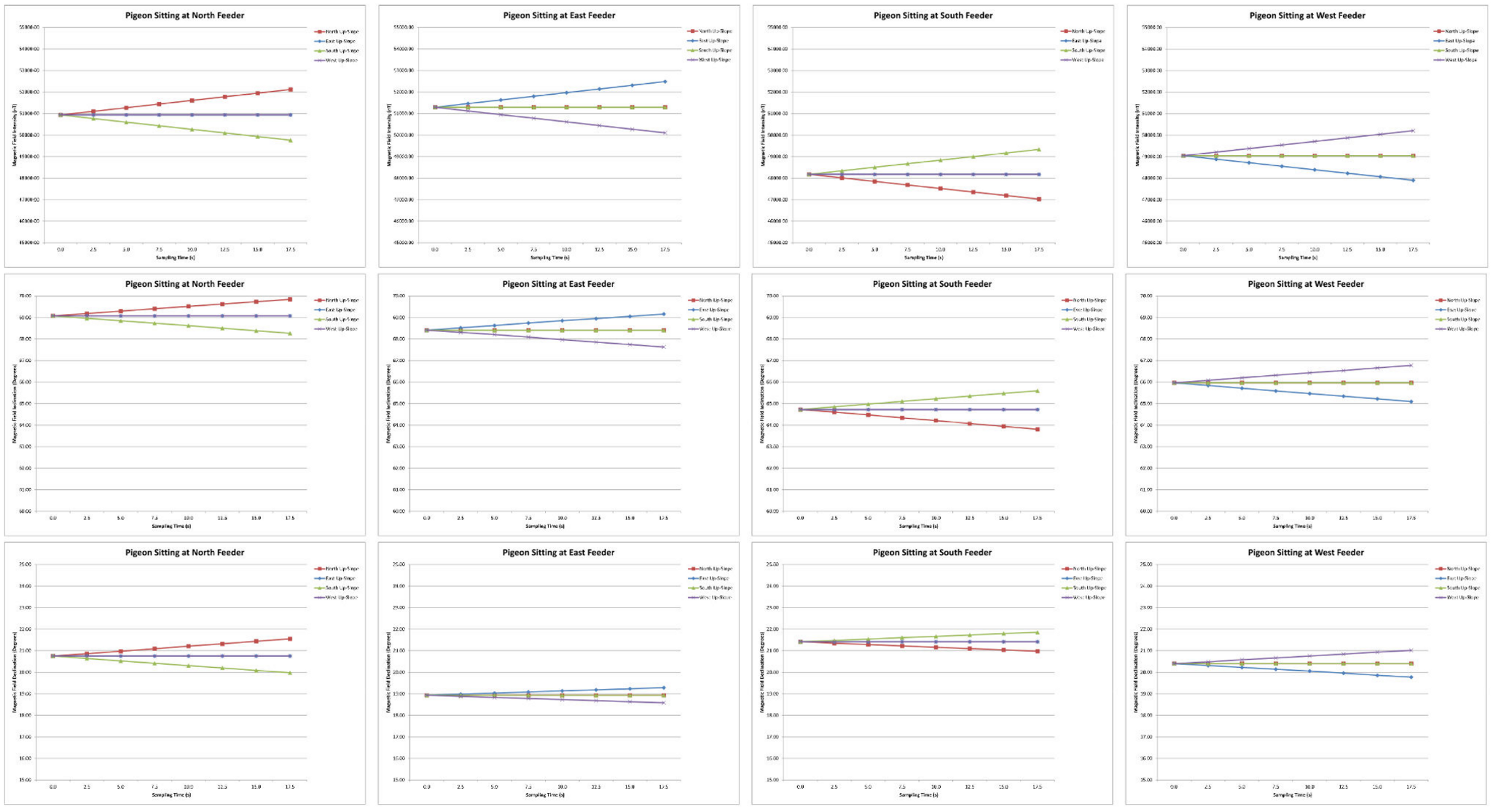

Supplement: Figure S2 — Magnetic field measurements during magnetic coils anti-parallel sessions. Magnetic field intensity (top row), magnetic field inclination (middle row), and magnetic declination (bottom row) experienced by pigeon sitting during 15 second sampling period in front of North feeder (first column), East feeder (second column), South feeder (third column), and West feeder (fourth column) for trials with the magnetic intensity gradient of the VMI-map being associated with either the North feeder (red), East feeder (blue), South feeder (red), or West feeder (purple). (TIF) [file pone.0072869.s002.tif]

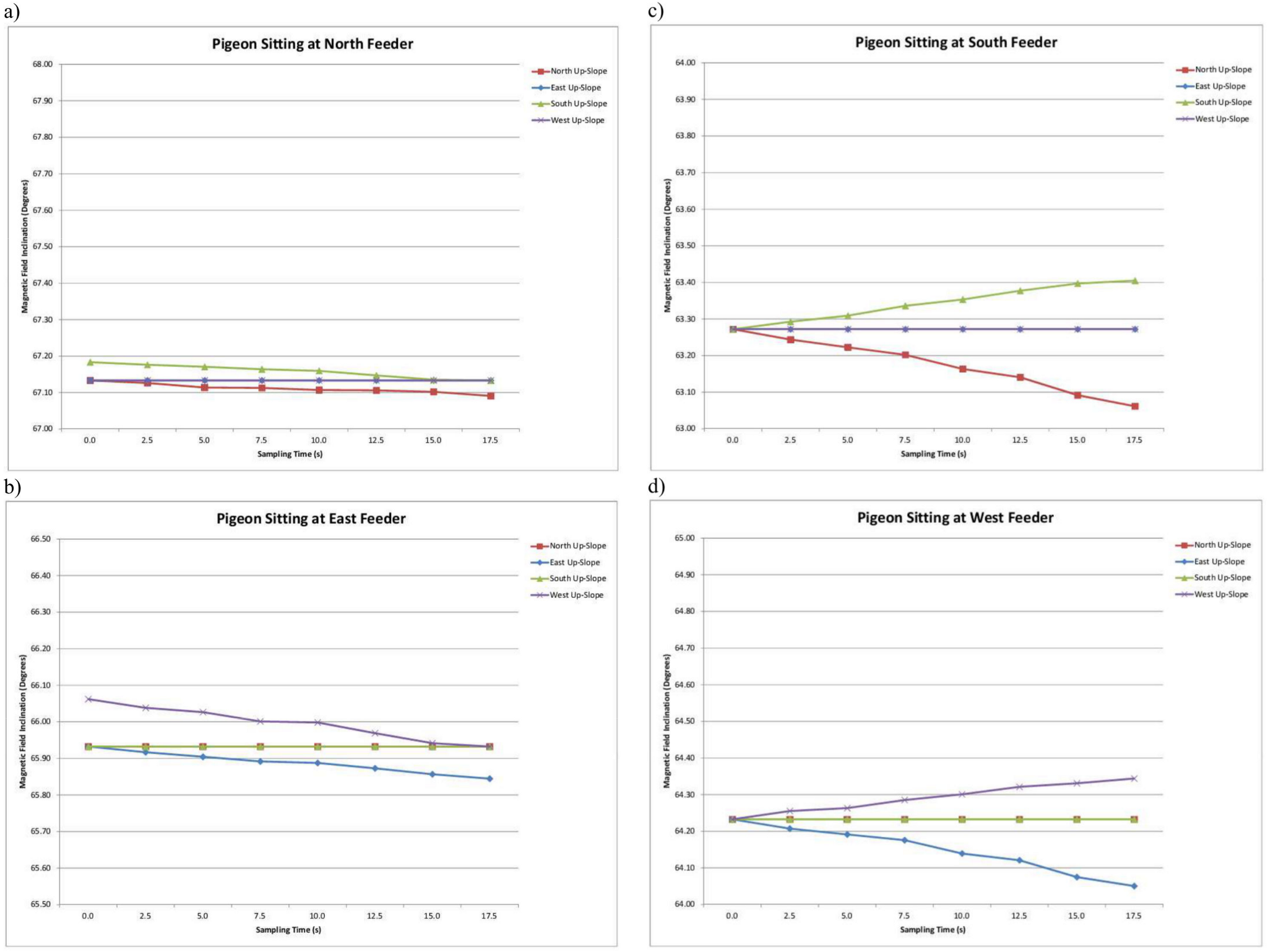

Supplement: Figure S3 — Magnetic field inclination measurements during magnetic coils parallel sessions. Magnetic field inclination experienced by pigeon sitting during 15 second sampling period in front of a) North feeder, b) East feeder, c) South feeder, and d) West feeder (fourth column) for trials with the magnetic intensity gradient of the VMI-map being associated with either the North feeder (red), East feeder (blue), South feeder (red), or West feeder (purple). Please note y-axis scale has been adjusted for each graph to show any inclination changes within 1/10th of a degree. (TIF) [file pone.0072869.s003.tif]

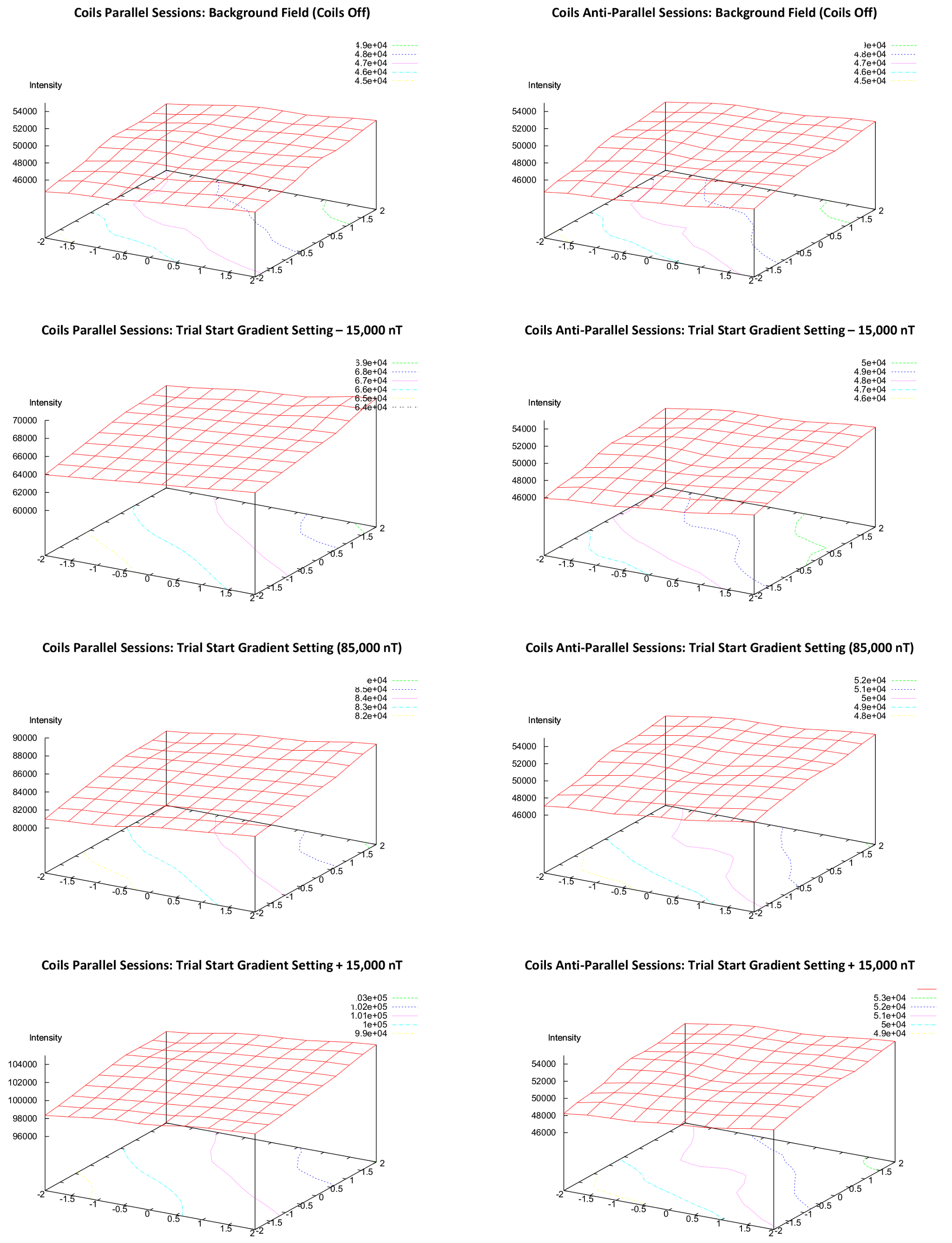

Supplement: Figure S4 — Background magnetic field intensity and magnetic field intensity generated by the coil system measured throughout experimental arena. The background field and the magnetic field parameters generated by the coil system were characterized with a FVM handheld 3-axis vector fluxgate magnetometer (Meda Inc.) at the head height of a walking pigeon at 25 points distributed throughout the experimental arena (center of arena, eight points at a distance of 15 cm from the center of the arena around the periphery of the arena at 45° intervals, 16 points at a distance of 30 cm from the center of the arena around the periphery of the arena at 22.5° intervals). Data points were then extrapolated and plotted as a meshgrid with the Splot function in GnuPlot 4.2 (patch level 3). The x- and y-axes show the location within the arena, with the center coordinate (0,0) being located at the center of the arena, and coordinates of 1.0 an 2.0 being representing 15 and 30 cm from the center of the arena respectively. The z-axis indicates magnetic field intensity. Measurements were made with the coils set to parallel (left column) or anti-parallel (right column) current flow with no current send to the coils (background field; top row) or with the VMI-software set either at the intensity gradient level for the trial start setting -15,000 nT (second row), the trial start setting (ca. 85,000 nT; third row), or the intensity gradient level for the trial start setting +15,000 nT (fourth row). (TIF) [file pone.0072869.s004.tif]

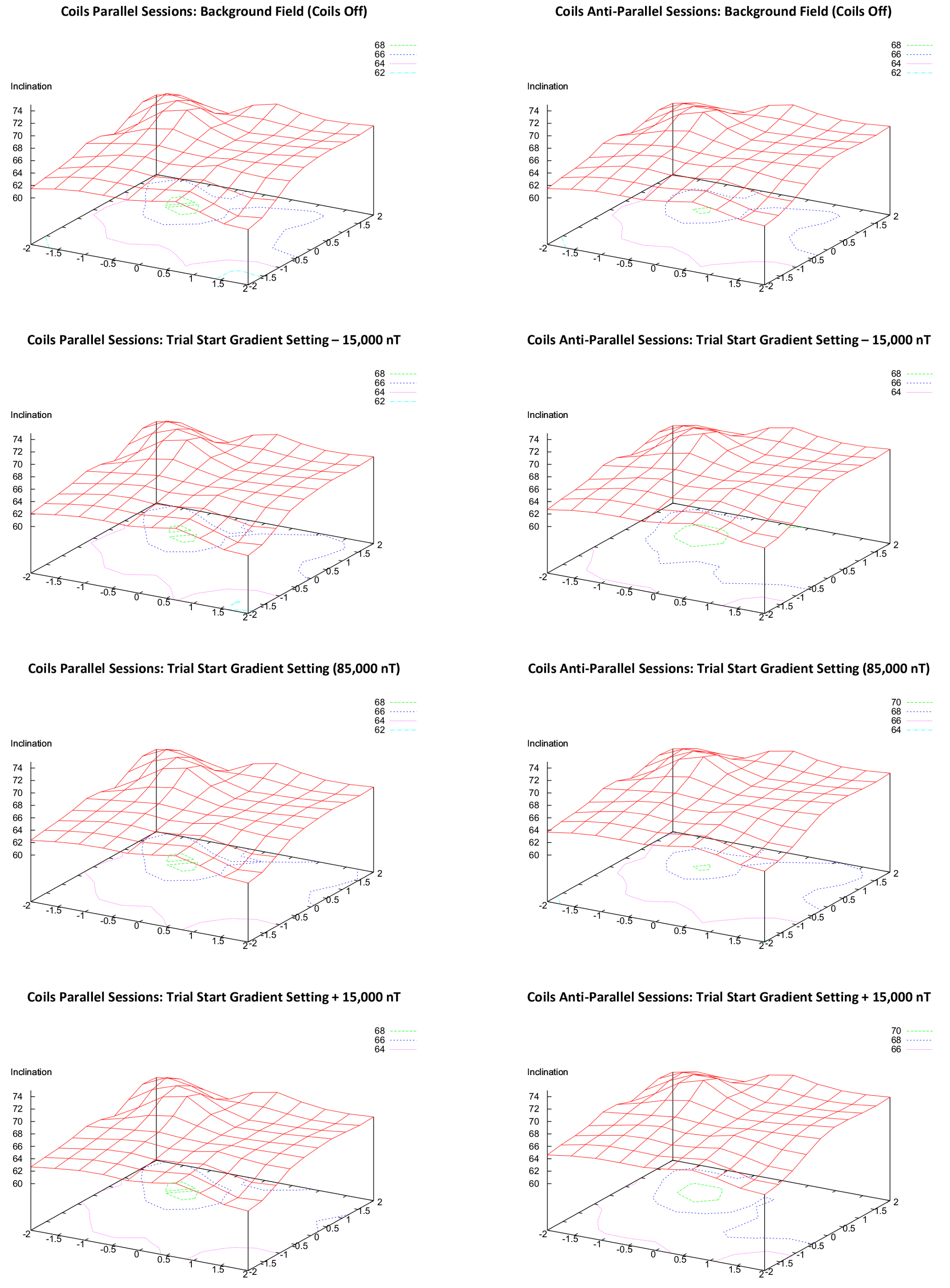

Supplement: Figure S5 — Background magnetic field inclination and magnetic field inclination generated by the coil system measured throughout experimental arena. The background field and the magnetic field parameters generated by the coil system were characterized with a FVM handheld 3-axis vector fluxgate magnetometer (Meda Inc.) at the head height of a walking pigeon at 25 points distributed throughout the experimental arena (center of arena, eight points at a distance of 15 cm from the center of the arena around the periphery of the arena at 45° intervals, 16 points at a distance of 30 cm from the center of the arena around the periphery of the arena at 22.5° intervals). Data points were then extrapolated and plotted as a meshgrid with the Splot function in GnuPlot 4.2 (patch level 3). The x- and y-axes show the location within the arena, with the center coordinate (0,0) being located at the center of the arena, and coordinates of 1.0 an 2.0 being representing 15 and 30 cm from the center of the arena respectively. The z-axis indicates magnetic field inclination. Measurements were made with the coils set to parallel (left column) or anti-parallel (right column) current flow with no current send to the coils (background field; top row) or with the VMI-software set either at the intensity gradient level for the trial start setting -15,000 nT (second row), the trial start setting (ca. 85,000 nT; third row), or the intensity gradient level for the trial start setting +15,000 nT (fourth row). (TIF) [file pone.0072869.s005.tif]

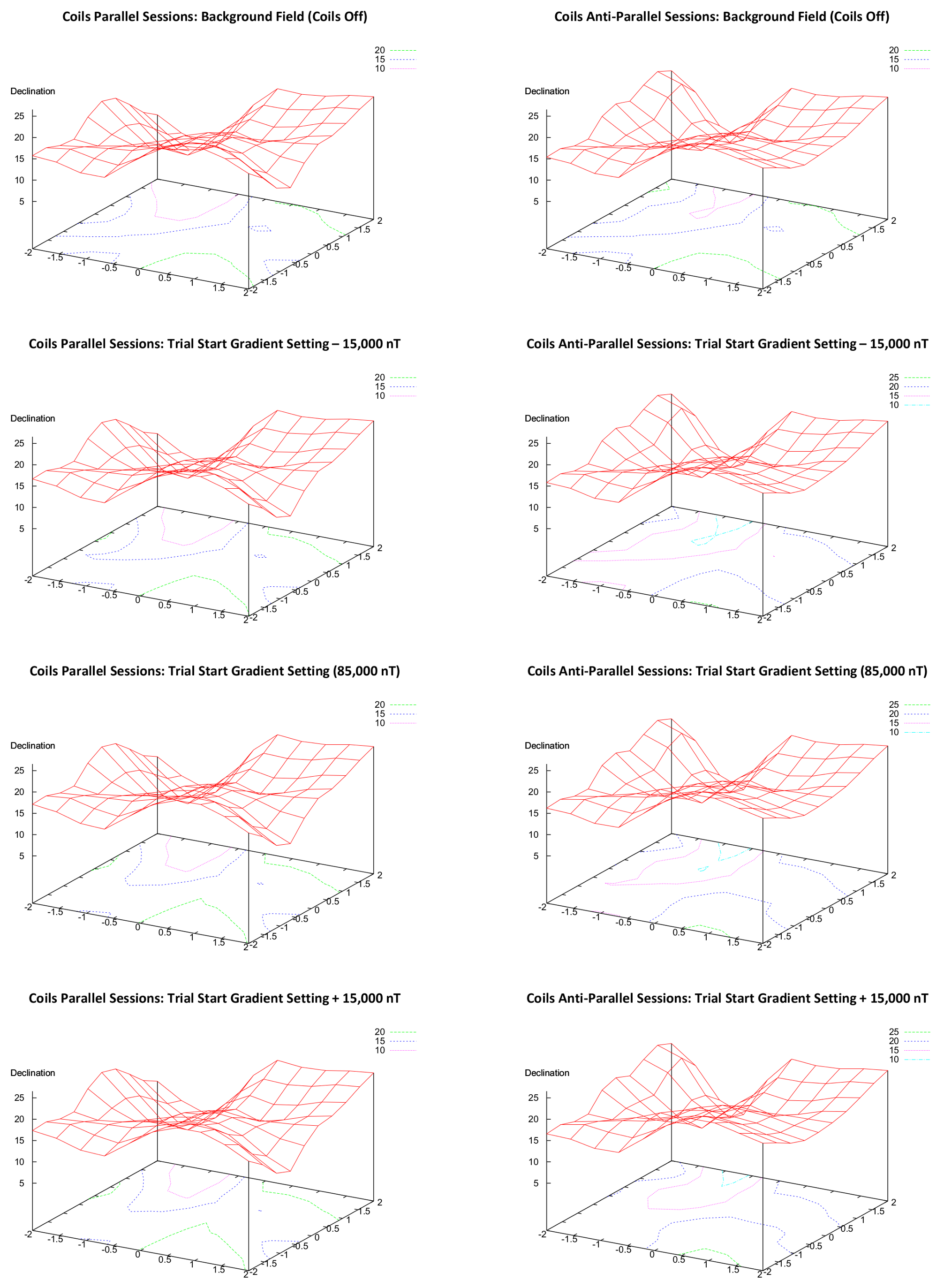

Supplement: Figure S6 — Background magnetic field declination and magnetic field declination generated by the coil system measured throughout experimental arena. The background field and the magnetic field parameters generated by the coil system were characterized with a FVM handheld 3-axis vector fluxgate magnetometer (Meda Inc.) at the head height of a walking pigeon at 25 points distributed throughout the experimental arena (center of arena, eight points at a distance of 15 cm from the center of the arena around the periphery of the arena at 45° intervals, 16 points at a distance of 30 cm from the center of the arena around the periphery of the arena at 22.5° intervals). Data points were then extrapolated and plotted as a meshgrid with the Splot function in GnuPlot 4.2 (patch level 3). The x- and y-axes show the location within the arena, with the center coordinate (0,0) being located at the center of the arena, and coordinates of 1.0 an 2.0 being representing 15 and 30 cm from the center of the arena respectively. The z-axis indicates magnetic field declination. Measurements were made with the coils set to parallel (left column) or anti-parallel (right column) current flow with no current send to the coils (background field; top row) or with the VMI-software set either at the intensity gradient level for the trial start setting −15,000 nT (second row), the trial start setting (ca. 85,000 nT; third row), or the intensity gradient level for the trial start setting +15,000 nT (fourth row). (TIF) [file pone.0072869.s006.tif]
